# Epithelial Ovarian Cancer and the Immune System: Biology, Interactions, Challenges and Potential Advances for Immunotherapy

**DOI:** 10.3390/jcm9092967

**Published:** 2020-09-14

**Authors:** Anne M. Macpherson, Simon C. Barry, Carmela Ricciardelli, Martin K. Oehler

**Affiliations:** 1 Discipline of Obstetrics and Gynaecology, Adelaide Medical School, Robinson Research Institute, University of Adelaide, Adelaide 5000, Australia; anne.macpherson@adelaide.edu.au (A.M.M.); carmela.ricciardelli@adelaide.edu.au (C.R.); 2 Molecular Immunology, Robinson Research Institute, University of Adelaide, Adelaide 5005, Australia; simon.barry@adelaide.edu.au; 3 Department of Gynaecological Oncology, Royal Adelaide Hospital, Adelaide 5000, Australia

**Keywords:** ovarian cancer, immune system, immunotherapy, checkpoint inhibition, adoptive T-cell therapy, tumour microenvironment, MUC16, glycans

## Abstract

Recent advances in the understanding of immune function and the interactions with tumour cells have led to the development of various cancer immunotherapies and strategies for specific cancer types. However, despite some stunning successes with some malignancies such as melanomas and lung cancer, most patients receive little or no benefit from immunotherapy, which has been attributed to the tumour microenvironment and immune evasion. Although the US Food and Drug Administration have approved immunotherapies for some cancers, to date, only the anti-angiogenic antibody bevacizumab is approved for the treatment of epithelial ovarian cancer. Immunotherapeutic strategies for ovarian cancer are still under development and being tested in numerous clinical trials. A detailed understanding of the interactions between cancer and the immune system is vital for optimisation of immunotherapies either alone or when combined with chemotherapy and other therapies. This article, in two main parts, provides an overview of: (1) components of the normal immune system and current knowledge regarding tumour immunology, biology and their interactions; (2) strategies, and targets, together with challenges and potential innovative approaches for cancer immunotherapy, with attention given to epithelial ovarian cancer.

## 1. Introduction

Recent advances in the understanding of immune function and the interactions with tumour cells have led to the development of various cancer immunotherapies and strategies for specific cancer types, prompting awarding the 2018 Nobel Prize in Physiology or Medicine for cancer therapy by negative immune regulation using checkpoint inhibitor antibodies [1]. Despite some stunning successes with US Food and Drug Administration (FDA) approved cancer immunotherapies for some patient groups, e.g., those with melanomas or lung cancer [2], overall results are variable and unpredictable. The majority of patients within these groups have derived little benefit [3].

Cancer immunotherapy can be defined as the treatment of cancer by inducing, enhancing, or suppressing the immune response [4], as well as repressing tolerance mechanisms, and can target either the cells of the immune system or the cancer cells. Observations of tumour infiltrating lymphocytes (TILs) that are associated with improved clinical outcomes in ovarian cancer, and recognition that it is an immunogenic disease [5,6,7], have made the concept of ovarian cancer immunotherapy increasingly attractive. However, it has been highlighted recently that immunotherapy for ovarian cancer is still in its infancy, [8], with only one monoclonal antibody therapy (bevacizumab), targeting angiogenesis in the tumour microenvironment (TME), being approved for its’ treatment [9]. Recent cancer immunotherapy advances tend to combine several agents and strategies, including anti-angiogenesis with more standard cancer treatments, for therapeutic synergy [10]. A detailed understanding of the biology and interactions between cancer and the immune system is vital for recognition and development of potential new immunotherapeutic strategies and targets as well as optimisation of existing immunotherapies, together with the application and modification of more conventional therapies for patients. This article reviews components of the normal immune system, current knowledge regarding tumour immunology and biology, strategies for and challenges of cancer immunotherapy, as well as potential innovative approaches that can be used together with immunotherapy, with a particular focus on epithelial ovarian cancer.

## 2. “Normal” Immune System Function

### 2.1. Innate and Adaptive Immunity

The immune system with innate and adaptive immune responses is responsible for detecting and destroying foreign invaders; while preventing responses against self-antigens (immune tolerance) to minimise tissue destruction and prevent autoimmunity. The innate immune system is more primitive in terms of evolution and includes phagocytes (macrophages and dendritic cells, DCs), natural killer (NK) cells, granulocytes (neutrophils, eosinophils, basophils, and mast cells), and the complement system [11]. Cells of this system respond to foreign invaders such as bacteria, viruses and parasites through the recognition of non-self molecular patterns, i.e., “pathogen-associated molecular patterns” (PAMPs) or endogenous danger signals or “damage-associated molecular patterns” (DAMPs), which are often based on mislocalised or foreign glycans (carbohydrate chains) [12]. The innate response is often fast for a new pathogen challenge but not very specific. The adaptive immune system provides a slower response time than the innate immune system, but is highly antigen specific and is mediated by T-cells (cell-mediated immunity) and B-cells (humoral immunity with production of antibodies). A key functional difference was thought to be that the adaptive immune system can generate immunologic memory [13]. Cell–cell contact between immune cells and their target cells provides an “immunological synapse” that is required for their activation of effector function [14], and this synapse can be blocked by tumour cells as part of immune evasion.

### 2.2. Antigen Recognition and Antigen Presenting Cells (APCs)

**Figure 1:**
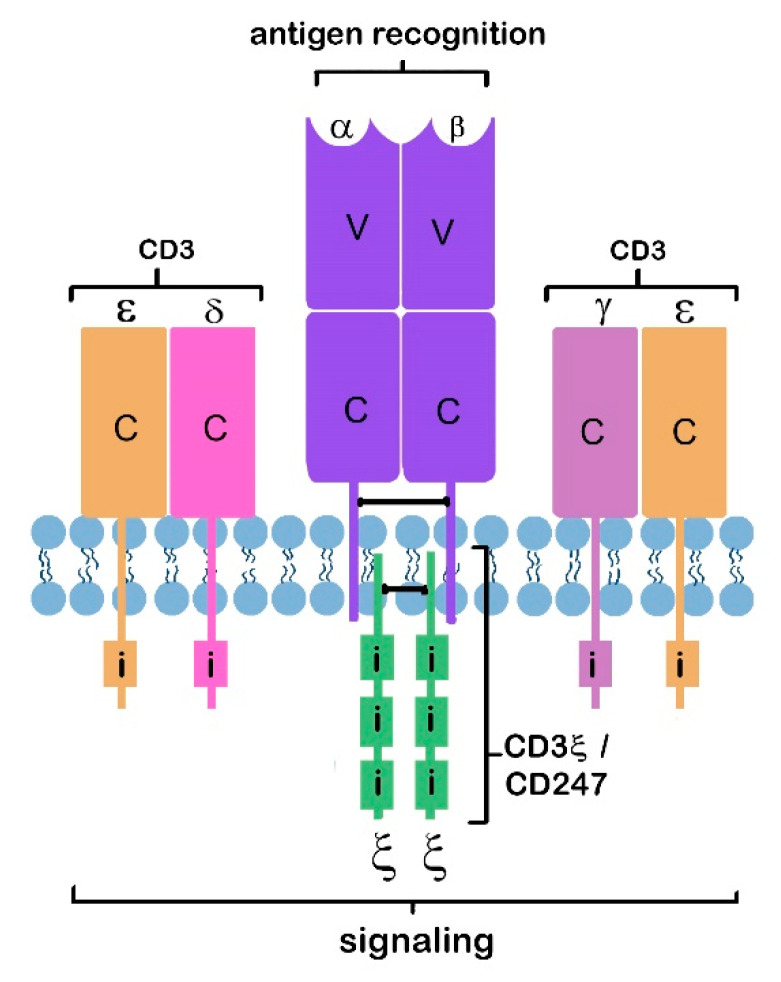
T-cell receptor (TCR) complex composed of α and β chains for antigen recognition, associated noncoavalently with CD3γε and CD3δε heterodimers, and a CD3ζ (CD247) homodimer. V, C = variable, constant immunoglobulin-like extracellular domains. i = ITAM (immune receptor tyrosine-based activation motif).

In the case of cell-mediated immunity, antigen recognition is “signal 1” in the path of activating or priming a naïve T-cell. This is enabled by the epitope-specific interaction between cell surface T-cell receptors (TCRs, glycosylated proteins mostly heterodimers of α and β chains) and antigen- presenting cells (APCs) which display antigen complexed with major histocompatibility (MHC) molecules—encoded by human leukocyte antigen (HLA) genes in humans—on their cell surface [15,16]. TCR diversity (and that of antibodies produced by B-cells in response to antigen) is determined by the random rearrangement of numerous gene cassettes that encode antigen receptor complexes on the cell surface of lymphocytes [17]. Following TCR engagement, signaling occurs through the CD3 protein complex consisting of three different nonpolymorphic polypeptide chains (heterodimers of ε and δ, and γ and ε; together with the homodimers of the CD247 protein—also known as CD3ζ (zeta)). Activation (signal 1) relies on the phosphorylation of immune receptor tyrosine-based activation motifs (ITAMs) [15]. Figure 1 shows the structure of the TCR complex, integral to T-cell function and the formation of an immunological synapse [14] at the cell surface interface between a T-cell and an APC.

APCs are classed as either professional or non-professional. Professional APCs (e.g., DCs, macrophages, B-cells), employ class II MHC molecules; with DCs and macrophages activated by interferon gamma (IFNγ), presenting foreign/exogenous antigen taken up by the APC through phagocytosis and presented to CD4+ helper (Th) 1 T-cells. Any nucleated cell in the body can behave as a non-professional APC and employs class I MHC molecules to display endogenous antigen derived from cytosolic proteins of self, tumour, or viral origin to CD8+ T-cells (effector T-cells, cytotoxic T-cells, killer T-cells) for sampling, which produce pro-inflammatory cytokines TNFα, IFNγ, and interleukin 2 (IL-2). Cross-presentation has an important role in immune surveillance by the innate immune system for infected cells and tumour cells, with subsequent regulation of the adaptive immune system with development of an appropriate cytotoxic T-cell response to these pathogens or malignancies [18,19]. Cross-presentation of exogenous antigen derived from tumour cells undergoing autophagy or immunogenic cell death, by MHC class I molecules on some DCs (conventional, cDCs) to naïve CD8+ T-cells in draining lymph nodes results in production of antigen-specific cytotoxic CD8+ cells [20,21,22]. Table 1 summarises the cell types and molecules involved in direct and cross-presentation of antigen.

### 2.3. Immune Checkpoints

For activation of naïve T-cells that have not previously been exposed to antigen, signal 1 is not sufficient on its own; full activation for T-cell priming requires a response and additional signals from the APCs [23]. Classically, this co-stimulation occurs via CD80 (B7-1) and/or CD86 (B7-2) at the cell surface of APCs with CD28 on the T-cell surface (“signal 2”) with phosphorylation of the TCR ITAMs and recruitment of intracellular second messengers. CD28 molecules respond to stimulation with production of an intracellular cascade of signals that enhance cytokine secretion (“signal 3”), including IL-2 and IFNγ, to defend against infection by inducing apoptosis of target cells [24] and prevent cellular anergy [25]. This T-cell activation promotes T-cell proliferation and cell survival via Bcl-XL transcription and the acquisition of killing capacity against target cells displaying the particular antigen via secretion of perforin and granzyme. B7 expression is limited under normal physiological circumstances, but is dramatically upregulated under inflammatory conditions [26]. Other T-cell surface costimulatory molecules include members of the tumor necrosis factor receptor superfamily (TNFRSF) CD27, OX40 (CD134), CD137 (4-1BB) and glucocorticoid-induced tumour necrosis factor receptor (GITR, CD357) which bind to their corresponding ligands members of the TNF superfamily (TNFSF), CD70, OX40L (CD252), CD137L, and GITRL (TNFSF18), on APCs [27].

The CD28 costimulatory signal also primes the immune system for regulation later in the course of the immune response by increasing T-cell expression of negative regulators [28]. This alternative decision, to suppress/tolerate rather than attack/stimulate the immune system, is also driven by the specific interaction between TCRs and displayed antigen from the APCs, and a negative co-stimulation molecule (signal 2) known as an immune checkpoint [13]. These T-cell immune regulating molecules include other members of the CD28 family, whose surface expression changes with different stages of T-cell differentiation and play a crucial role in modulating the T-cell response. These include: CTLA-4 (cytotoxic T-lymphocyte-associated protein 4, CD152), ICOS (inducible T-cell costimulatory; CD278), PD-1 (programmed cell death 1 encoded by gene PDCD1, also CD279 protein), and BTLA (B- and T-lymphocyte attenuator) [29]. Their respective binding partners expressed on APCs, are CD80 (B7-1) or CD86 (B7-2), B7-H2 (ICOSL, CD275), programmed death ligand-1 (PD-L1, B7-H1, CD274) or PD-L2 (B7-DC, CD273), and HVEM (Herpes virus entry mediator). Table 2 summarises immune checkpoint interactions and effects (stimulatory or inhibitory) on T-cells or other immune cells.

### 2.4. Mechanisms of Tolerance

Under normal physiological conditions, immune tolerance is required for self-antigens to prevent autoimmunity (central tolerance), antigens in the gut and liver derived from the microbiome and food (peripheral tolerance), and for non-rejection of the semi-allogenic fetus by the maternal immune system [45]. Immune tolerance is normally effected by immune editing that occurs centrally in the thymus and bone marrow for T-cells and B-cells, respectively, with deletion of autoreactive lymphocyte clones by apoptosis (negative selection), or induction of anergy. This is a hyporesponsive state with non-reaction by lymphocytes to a specific antigen resulting in diminished proliferation and IL-2 production. It can occur in response to TCR engagement by antigen without subsequent adequate CD28 co-stimulation (i.e., signal 1 without signal 2), and/or high co-inhibition [10,46].

CTLA-4 delivers an inhibitory signal from activated T-cells in secondary lymphoid organs and eliminates autoreactive T-cells in lymph nodes early in the immune response [28]. It does this by outcompeting CD28 and binding the same CD80 or CD86 molecules on APCs or haematological tumour cells with higher affinity and avidity than CD28 [47,48,49], so blocking the activating signal 2. It is expressed predominantly on CD4+ and not CD8+ CTLs [50]. CTLA-4 function results in dampening activation of effector CD8+ T-cells as well as driving the suppressive function of CD4+ regulatory T-cells (Tregs) to maintain immune tolerance [50]. Tregs have strong expression levels of CTLA-4 [51]. PD-1 regulates previously activated T-cells in non-lymphatic tissues, including the TME, later in the immune response and controls apoptosis of Tregs [52,53]. Engagement of PD-1 with PD-L1 on APCs delivers inhibitory signals and contributes to tolerance [28], as does engagement of PD-L1 on APCs or stromal cells with CD80 (the canonical costimulatory ligand on APCs) on T-cells [29].

CD4+ FOXP3+ CD25+ Tregs are the subpopulation of CD4+ T-cells specialised in the suppression of immunopathogenic responses from the host immune system against self or foreign antigens, and contribute to inhibition of autoimmunity and resolution of productive effector T-cell responses. Loss of Treg function promotes autoimmunity, while enhanced function contributes to carcinogenesis. Tregs use multiple mechanisms to suppress the proliferation of any cytokine-secreting effector T-cell, including cytokine sequestration, as they constitutively express IL-2 receptor alpha (IL-2Rα, CD25), CTLA-4, GITR (CD357), and OX40 (CD134) [54]. Together with Tregs, DCs are vital for maintaining central and peripheral tolerance by the delivery of co-stimulatory/co-inhibitory signals and cytokines that determine appropriate effector or Treg responses [55].

Abundant terminal sialic acid residues on glycans provide another mechanism of immune modulation that suppresses immune activation against the healthy self [56]. Sialic acids constitute a family with over members of 9-carbon acidic sugars, based on neuraminic acid [57]. Most white blood cells express cell surface sialic acid-binding immunoglobulin-like lectins (Siglecs) [58]. Many Siglecs are inhibitory due to their inclusion of immune receptor tyrosine-based inhibitory motifs (ITIMs) on their cytoplasmic tail which may be phosphorylated, thereby dampening immune cell signaling [56].

## 3. Ovarian Cancer Biology

Ovarian cancer is a heterogeneous group of malignancies with poor overall survival due to late stage diagnosis and limited treatment response due to progressive development of chemoresistance. Consequently, the five-year overall survival for advanced stage disease is only approximately 25–35% [59]. Epithelial ovarian cancers constitute more than 90% of ovarian malignancies, and include high-grade serous carcinoma (HGSOC), low-grade serous carcinoma (LGSOC), endometrioid carcinoma, mucinous carcinoma, and clear cell carcinoma. Of these, HGSOC is the most common histological subtype [59]. Ovarian cancer subtypes have also been grouped into type 1 and type 2 tumours [60]. Type-I tumors include endometrioid, clear cell, LGSOC, mucinous carcinomas and contain genetic changes including BRAF and KRAS mutations, whilst type-II tumors encompass HGSOC, carcinosarcoma, undifferentiated carcinoma, and primary peritoneal carcinoma [60]. Type-II tumors are characterised by TP53 mutations, widespread genomic instability, and initial sensitivity to platinum-based chemotherapy [61,62]. Efforts have sought to identify different HGSOC molecular subtypes with varying survival, clinical and tumour pathology characteristics [63,64]. An immune signature defines a subgroup of HGSOC with a high proportion of infiltrating lymphocytes with better survival outcome; whilst a reactive stromal signature with high levels of desmoplasmia, activated myofibroblasts, vascular endothelial cells, and extracellular matrix remodelling predicts the poorest prognosis [63,64]. HGSOC was long thought to arise from transformed surface ovarian epithelial cells, but studies have suggested that a large number of cases arise from the fimbriated end of the fallopian tubes [65,66,67].

## 4. Tumour Microenvironment (TME) of Solid Cancers Including Ovarian Cancers

As mentioned above, cancer cells together with the surrounding non-transformed cells create a TME whose composition is a major determinant of cancer progression. A network of cytokines, growth factors, inflammatory mediators and matrix remodelling enzymes drives communication within the TME [25]. Evolution of the TME has parallels with the microenvironment formed during wound healing [68]. However, cancers resemble wounds that fail to heal and promote a state of chronic inflammation, with high levels of reactive oxygen species (ROS), cytokines, chemokines, and growth factors [69]. Normal wound healing involves local lymphocyte infiltration, tissue degradation, phagocytosis of debris, secretion of cytokines and proteases, alteration of tissue architecture and ECM, followed by activation and proliferation of stromal fibroblasts and endothelial cells to restore tissue homeostasis [70]. Inflammation is an important driver of the wound healing response as well as cancer [70]. The tissue remodelling process is tightly regulated and limited in wound healing, but with cancer the self-limitation is absent [25].

Ovarian cancer metastasizes very early in the disease process. Malignant cells shed from the primary tumour, survive anchorage-independent apoptosis as free-floating cells or form spheroids, and spread throughout the peritoneal cavity to proliferate and interact with mesothelial cells and adipocytes of the omentum [71,72]. Ovarian cancer cells preferentially home to “milky spots” of the peritoneum [73], aggregates of immune cells, including macrophages, lymphocytes, and plasma cells supplied by blood and lymphatic vessels, which function as secondary lymphoid organs and promote immunity to peritoneal antigens [74]. The peritoneal environment also includes the peritoneal serous surface epithelium (mesothelial cells), underlying basement membrane and subperitoneal stroma that is composed of an extracellular matrix (ECM), fibroblasts, blood vessels, lymphatics and nerve fibres, and few blood-derived cells in the normal state. In ovarian cancer these elements together contribute to a pro-inflammatory ovarian cancer TME [75]. A study has shown that the degree of peritoneal metasteses in patients with (epithelial) ovarian cancer is associated with patient age [76].

The non-tumour cellular component of the TME in ovarian cancer includes a variety of immune cells comprising myeloid lineage derived cells including myeloid-derived suppressor cells (MDSCs), tumour associated macrophages (TAMs), tumour-associated neutrophils (TANs), and other lymphoid lineage derived cells including TILs. These immunosuppressive cells produce inhibitory cytokines such as IL-4, IL-10, LIF, and TGFβ that repress T-cell function [77]. Non-immune cells such as mesothelial cells, adipocytes, cancer-associated fibroblasts (CAFs), and ECs are also important constituents of the ovarian cancer TME that modulate the actions and clinical impact of immune cells [78,79].

### 4.1. Immune Cellular Component of the TME

#### 4.1.1. TAMs and MDSCs

TAMs are the most abundant immune cell population present in tumour tissue. TNFα is constitutively expressed in macrophages and ovarian cancer cells of the TME [80,81], and is a major mediator of cancer-related inflammation [82]. In ovarian cancer, TAMs expressing B7-H4 (a member of the B7 superfamily; V-set domain containing T cell activation inhibitor 1, [83] gene) suppress the activation of antigen-specific T-cells; and CCL22 secreted by TAMs recruits CCR4-expressing Tregs and promotes tumour growth [84]. MDSCs are a mixed population of precursors of dendritic cells, macrophages and granulocytes at various stages of differentiation that are stimulated by tumour-derived factors, are potently immunosuppressive, and contribute to tumour progression [72,85]). They are found in some malignancies including ovarian, breast, colon, pancreatic and non-small cell lung cancer, and are associated with escape from host immunity and poor outcomes [86,87]. M1 and M2 macrophages were significantly associated with better outcomes in patients with high-grade type-II ovarian cancer but worse outcomes amongst patients with type-I ovarian cancer [88].

#### 4.1.2. TILs

TILs include CD8+ T-cells, Tregs, regulatory B-cells (Bregs), type II NK T-cells (which recognise lipid antigens and are not the same as NK cells), and Th2 CD4+ cells which upregulate B-cell responses, play roles in remodeling and may or may not promote tumour growth [68,71]. They can mediate immune editing of cancer cell by processes of elimination, equilibrium and escape [89]. Although tumour-associated immune cells of the TME can initially play an important role in tumor growth restriction, they are also immunosuppressive and promote tumour progression via their ability to suppress host anti-tumour responses and stimulate tumour angiogenesis [86]. The presence of immune infiltrate in cancers has been correlated with a favourable prognosis following treatment in a range of malignancies including ovarian, breast and colorectal cancers as well as melanomas [90]. The five-year survival rate with epithelial ovarian cancer was found to be increased eight-fold with the presence of TILs, from 4.5% to 38.0% [5]. A higher ratio of CD8+ lymphocytes to Tregs (CD4+ CD25+ FOXP3+) in the TILs of epithelial ovarian cancer tissues is associated with a more favourable prognosis [91]. A meta-analysis with almost 4500 patients with mixed or serous histologic subtypes of ovarian cancer showed that intraepithelial CD3+, CD8+, and CD103+ TILs are clearly associated with increased overall survival and disease specific survival, with the location of TILs within the tumour being important for this prognostic effect [7]. T-cell infiltration has been shown to be subtype dependent in ovarian cancer [92,93,94]. Several studies have found that activated CD4+ T or CD8+ T tumour infiltrating lymphocytes are associated with good overall survival in HGSOC [93,94,95,96,97]. Different T-cell infiltration is prognostic for other epithelial ovarian cancer subtypes. A 2018 study only found CD4+ T-cells to be significantly associated with overall survival in LGSOC [93], while the presence of intraepithelial CD8+ T-cells was not associated with improved survival in endometrioid or clear cell carcinomas [95]. A subgroup of clear cell carcinomas with microsatellite instability where found to have increased CD8+ TILs, a higher CD8+/CD4+ ratio, and higher PD-1+ TILs [92]. Together, these findings indicate that ovarian cancer subtype also needs to be considered in the development of immunotherapies.

CD8+ T-cells are the major anti-tumour effector cells, and many cancer immunotherapeutic approaches seek to amplify cytotoxic T lymphocytes (CTLs) specific to tumour cells [98]. CD103 (ITGAE, integrin αE) appears to be a definitive marker for antigen-experienced tissue resident memory T-cells (Trm) CD8+ cells [99], a subpopulation of effector memory cells with cytotoxic properties that circulate through various lymphoid and non-lymphoid tissues including the skin, gastrointestinal tract, lung, and genitourinary tracts [98,100]. Differentiation towards a residency memory phenotype is regulated by TGFβ and IL-15 [98,100]. The ligand for CD103 is E-cadherin, which is found on the surface of epithelial and tumour cells and facilitates tumour cell recognition and adhesion [99,101]. Following TCR engagement, the Trm cell undergoes lytic granule polarisation and degranulation [99]. CD8+ CD103+ TILs have been shown to express high levels of PD-1 in various cancers, including HGSOC and melanoma [100]. Evidence suggests that CD103 plays an important role in protective immunity against cancers of epithelial origin in TILs in ovarian cancer and melanoma, and the number of CD8+ CD103+ cells increase in response to treatment with anti-PD-1 in mice and humans [100]. The apparent paradox of increased PD-1 expression associated with increased patient survival, when PD-1 is known to be an inhibitor of immune response and seen to be a marker of T-cell terminal differentiation and exhaustion, can be explained by functional adaptation by T-cells with concurrent maintenance of effector function for tumour control and minimal bystander tissue damage [102]. Expression of PD-1 and LAG-3 (lymphocyte-activation gene 3, a negative immune checkpoint regulator found on Tregs that binds MHC class II) are commonly co-expressed on anergic or exhausted T-cells [49]. Both of these checkpoint inhibitors are highly enriched in CD103+ tumour-resident CD8+ T-cells in untreated metastatic melanoma patients, suggesting that these cells are likely to be the initial target of anti-PD-1 treatment. Expansion of these cells was found in biopsy specimens from advanced stage metastatic melanoma patients being treated with anti–PD-1 therapy (nivolumab, pembrolizumab) [103]. These and other results suggest that immune profiling and enrichment of CD103+CD8+ T-cells will be useful for devising successful strategies for immunotherapy [100,103].

Tregs secrete granzymes (serine proteases) and perforin to impair the function of effector T-cells (i.e., Th cells and CTLs) and NK cells, as well as immunosuppressive factors such as IL-10 or TGFβ [104]. They also express CD25 (a component of the IL-2 receptor) at their cell surface, and due to their high avidity for IL-2 create a sink for this T-cell growth factor [105]. CD8+ CD25+ FOXP3+ Tregs have been found in patients with ovarian, prostate or colorectal cancer where they exert similar immunosuppressive effects [106,107].

B-cells have also been found as components of TILs within the TME of ovarian [108] and other cancers [109]. Like T-cells they contribute tumour suppressing, effector-cell subtypes; and immunosuppressive, regulatory B-cells (Bregs) [109]. Effector B-cells secrete antibodies, present antigen to T-cells and promote T-cell responses, and can directly kill cancer cells via granzyme B [109]. Bregs, producing IL-10, and expressing high levels of CD80 and CD86, also contribute to the maintenance of tolerance and immunosuppression. They inhibit CD4+ and CD8+ T cell proliferation and promote the conversion of CD4+ T-cells into suppressive Tregs [110]. The balance of B-cell subtypes within the TME is likely to affect overall tumour progression and outcome. Ascites from ovarian cancer patients has been found to be enriched with a population of IL-10 producing Bregs that suppresses IFN-γ production by CD8+ T-cells [111].

#### 4.1.3. NK Cells

NK cells are now regarded as bridging the innate and adaptive immune systems since (like T-cells), they can gain memory functional phenotypes after encountering target cells [112]. Like CD8+ T-cells, the normal function of NK cells is to kill infected or transformed cells by release of perforin, granzymes, and TNF family members, though their mechanisms of target recognition and subsequent signaling cascades is different [113]. The function of NK is modulated by a range of inhibitory and activating receptors that allows them to sense and respond to their environment such as changes in expression of ligands of pathogenic cells, and loss of MHC in tumour cells [112]. They are activated by endogenous ligands that are upregulated in response to stress (e.g., from viral infection, DNA damage, or TNF signaling), as well as inflammatory cytokines and immunoglobulin Fc [114]. NK activating receptors include CD16, NKp30 (gene NCR3, natural cytotoxicity triggering receptor 3), NKG2D [112]. NK cells are inhibited by MHC proteins that are highly expressed on healthy cells and engage NK cell inhibitory receptors e.g., killer-cell immunoglobulin-like receptors (KIRs), and the CD94-NKG2A heterodimer to prevent NK cell activation [114]. NK cells are present in relatively high numbers in the ascites of patients with ovarian cancer, but display functional impairment with downregulation of CD16 and reduced antibody-dependent cell-mediated and natural cytotoxicity [115]. Tumour cell release of soluble forms of activating NK cell receptor ligands may limit surface expression of the activating NK receptors, thus affecting the ability of NK cells to kill tumour cells that express ligands for those receptors. Ovarian cancer cells release a soluble form of B7-H6 (gene NCR3LG1, natural killer cell cytotoxicity receptor 3 ligand 1), the main ligand for NKp30 present on NK cells, leading to the loss of NKp30 expression on NK cells in the TME [115]. These NK cells display impaired IFN-γ production and cytolytic function, with subsequent poor NK cell-mediated elimination of B7-H6+ ovarian cancer cells [115].

#### 4.1.4. Dendritic Cells

Optimal DC function is required for protective adaptive T-cell–based responses against tumours. [116]. Inhibition of normal DC activation, maturation and function, with promotion of a tolerogenic DC phenotype (i.e., poor capacity for antigen presentation or reduced expression of co-stimulatory molecules [116]) is a key mechanism of tumour immune evasion. This DC phenotype is driven by tumour secretion of VEGF-A, TGFβ, and IL-10, and aided by hypoxia and lactic acid production in the TME [117]. Severe DC dysfunction occurs in the TME of advanced ovarian cancers, with massive infiltration of DCs and secretion of PGE2 and TGF-β by cancer cells and transformation of immunocompetent conventional DCs into immunosuppressive cells via the induction of PD-L1 and arginase activity [116].

### 4.2. Non-Immune Cellular Component of the TME

#### 4.2.1. Mesothelial Cells and Adipocytes

The mesothelium lining of the peritoneum is a cobblestone monolayer of cells with characteristics of both epithelial and mesenchymal cells that normally act as a barrier and secrete factors to maintain peritoneal homeostasis [118]. The aged peritoneum accommodates increasing numbers of senescent mesothelial cells that secrete various factors (e.g., fibronectin, ICAM-1, beta-galactosidase, and angiogenic and inflammatory mediators) into the ovarian cancer TME that enhance cellular adhesion and tissue stiffness [76,118]. Senescent mesothelial cells also induce ovarian cancer cell production of angiogenic factors, and promote migration and invasion [119]. Adipocytes constitute most of the omentum and produce cytokines to foster cancer growth and metastasis, and can alter their lipid metabolism to provide fatty acids to cancer cells as a fuel source for rapid growth [73].

#### 4.2.2. CAFs

CAFs are phenotypically similar to activated fibroblasts found in wound healing tissue. They are derived predominantly from resident fibroblasts, however they can also arise by trans-differentiation of other cell types (e.g., epithelial, endothelial, pericytes, adipocytes, and bone marrow derived mesenchymal stem cells) in response to tumour derived factors [72]. These include TGFβ, PDGF-BB, bFGF, VEGF, microRNAs, reactive oxygen species (ROS), MMPs, and extracellular vesicles (EVs) [72]. CAFs influence immune cell recruitment and differentiation, and can acquire a pro-inflammatory signature with expression of TGFβ, PD-L1/L2, and chemokines that can promote recruitment of immunosuppressive myeloid cells [78]. They are responsible for ECM growth in the TME and remodelling that contribute to desmoplasia and stromal stiffness that is associated with poorer patient outcome [63,120,121]. Increased levels of the transcription factor SNAI2 protein present in stromal fibroblasts from ovarian cancer patients with a desmoplasia subtype may provide a future therapeutic target [120]. CAFs and the epithelial-mesenchymal transition (EMT) contribute to the invasive and metastatic abilities of ovarian cancer cells, and the different forms of ovarian cancer (low and high grade serous, mucinous, clear cell) have been shown to contain distinct populations of CAFs [122]. Invasive and chemoresistant spheroids present within ascites of HGSOC patients, are formed between fibroblast activation protein positive (FAP^+^) CAFs that recruit integrin α5 (ITGA5) positive ascitic tumour cells, and are fundamental to metastatic dissemination [123]. In contrast, LGSOC which has a more favourable outcome than HGSOC with delayed metastasis, has a relative lack of CAFs [123]. In addition, ovarian cancer-derived EVs are able to activate normal fibroblast cells in vitro [124].

#### 4.2.3. Endothelial Cells and Tumour Vasculature

Endothelial cells (ECs) form the inner cellular monolayer of blood and lymphatic vessels, and so provide the interface for transendothelial migration (diapedesis) of lymphocytes into the surrounding tissue, with precise spatiotemporal control due to co-evolved biochemical information exchange [125]. They are also now recognised as “semi-professional” antigen presenting cells [125]. In the pro-inflammatory TME, damaged cells release DAMPs that translocate to the cell surface and are then sensed by pattern recognition receptors (PPRs; such as toll-like receptors (TLRs) and nucleotide-binding oligomerisation domain-like-receptors) present on various leukocytes. The resultant release of cytokines and chemokines including TNFα, VEGF, interleukins and histamine further enhances inflammation and stimulates angiogenesis (new vessel growth from pre-existing vessels) with proliferation and migration of ECs together with their activation to promote leukocyte diapedesis [126]. Tumour angiogenesis is a dysregulated process, with resulting vessels being structurally abnormal with EC junctional defects, tortuous, dilated, hyperpermeable, poorly covered by pericytes, and with patchy perfusion [127,128]. The hypoxic TME and high metabolic rate and demand for constant nutrient supply of tumours, contributes to deregulated vascular processes in ovarian cancer. Ovarian tumour vasculature may be formed not only from ECs early in the disease process (angiogenesis), but also from vessel co-option, vasculogenesis and vasculogenic mimicry [129]. During vasculogenic mimicry, cancer stem cells transdifferentiate into endothelial-like cells that form looped capillary-like structures with extravascular patterned matrix rich in laminin, collagen type IV and VI and glycosaminoglycans particularly heparin sulphate proteoglycan (HSPG), capable of transporting plasma and red blood cells [130]. Vasculogenic mimicry has been shown to increase in a preclinical model of ovarian cancer in response to treatment with bevacizumab [131], although the chosen cell line (SKOV3) is unlikely to be representative of HGSOC [132]. Vasculogenesis, in which ECs are derived from in situ differentiation of myeloid cells or endothelial progenitor cells, is an important tumour process when the angiogenic pathway is blocked e.g., in response to anti-angiogenic or radiotherapy treatment [129].

High endothelial venules together with immune aggregates that recruit naïve T- and B-cells from the circulation, form tertiary lymphoid structures similar to those normally present in lymph nodes, and are present in various cancers including ovarian, where they can support an adaptive anti-tumour immune response [78,133].

## 5. Cancer and Immune System Interactions

Hallmarks of cancer have been recognised for some time and include growth stimulation, evasion of growth suppressors, resistance to apoptosis, replicative immortality, induction of angiogenesis, and activation of invasion and metastasis; with reprogramming of metabolic pathways and evasion of the immune system being added to the list more recently [134].

### 5.1. Tumour-Associated Antigens (TAAs)

In the early stages of cancer, tumour cells are likely to be antigenically identical to their normal counterparts, hence tolerated as self-tissues, which can be mediated by the recruitment of Tregs to the TME. As cancers progress, they accumulate mutations and express de novo tumour associated antigens (TAAs) on their surface [17,135]. Rapid tumour growth often results in local hypoxia, and together with accumulating mutations that compromise cell viability, leads to tumour necrosis and release of TAAs [17]. This signals recruitment of macrophages or dendritic cells to the area where they phagocytose the debris and act as professional APCs. CD4+ T helper cells and CD8+ CTLs may then recognise the resulting peptide-MHC complexes presented to their antigen-specific TCRs [17].

TAAs may be abnormal proteins (neoantigens, whose presence is greatest in immunologically hot tumours with a high mutational load [136]), or normal proteins that are not usually expressed in an adult (differentiation antigens), or overexpressed antigens that are normally expressed at low levels. The cancer-testis antigens (CTAs) are a large family of tumour antigens widely expressed by tumours including ovarian cancer, with expression in healthy tissues limited to normal placenta and testis [137]. CTAs are normally involved in self-renewal and differentiation of pluripotent and multipotent stem cells, while aberrant expression is associated with cancer transformation and abnormal differentiation of cancer stem cells [138]. Studies have implicated global and locus-specific DNA hypomethylation as a key mechanism promoting CTA expression in cancer, including epithelial ovarian cancer [139]. Cancer patients often develop spontaneous immune reactions against CTAs demonstrating their immunogenicity [137]. CTA family members in ovarian cancer reported to date include MAGE genes, NY-ESO-1, SSX, and CT45, which are located on the X chromosome, and BORIS, PRAME, PIWIL, and AKAP3/4, which are categorised as non-X chromosome CTAs [140]. NY-ESO-1 (product of the CTAG1B gene) is perhaps the best characterised member of this family [137], and may be selectively expressed by cancer stem cells and is expressed in the tumours of approximately 40% of ovarian cancer patients [6].

EGFR and HER2 are examples of overexpression/gene amplification of antigen, which is a frequent event in many cancers, predominantly in cancers of epithelial origin [141], and is associated with poor prognosis. HER2, (human epidermal growth factor receptor 2; also known as Neu and ERBB2), is a member of a family of four different epidermal growth factor receptors (EGFRs) present on the cell surface of epithelial cells. HER2 has the strongest tyrosine kinase activity of the family, and exists in an activated conformation without any ligand binding activity [142]). Estimates of HER2 overexpression in ovarian cancer varies, with values ranging between 22–66%, with a contribution of genomic amplification in 11% [143], and 6–30%, with an estimate of only 3% for serous ovarian cancer [142]. EGFR overexpression has been estimated to be present in 30–70% of ovarian cancer cases [142]. The EGFR pathway is thought to have a role in the acquisition of resistance to anti-VEGF therapy, and treatment with anti-EGFR can select for tumour cell subpopulations with increased angiogenic potential [80].

MHC-II may be expressed by many other cell types besides professional APCs, including some tumour cells. Ovarian cancer cells are capable of expressing MHC-II related genes, often in response to inflammatory signaling processes induced by high levels of IFNγ in the TME. Tumour-specific MHC-II expression may increase recognition of a tumour by the immune system, and may therefore play an important role in immune recognition. It has been associated with favourable prognosis, improved response to immune checkpoint inhibition in humans and increased tumour rejection in murine models [144].

### 5.2. Immunosuppression

Initially, CD8+ CTLs and CD4+ T helper cells can limit cancer development by production of IFNγ and cytotoxins, but many mechanisms including an immunosuppressive TME and chronic inflammation can override these effects to promote cancer development [145]. Failure of an effective antitumour immune response can occur through inadequate processing of TAAs by dendritic cells or poor presentation of antigens to their antigen-specific T-cells, or lack of maintenance of the T-cell response for a sufficient time period to eliminate the cancer. To be effective, activated T-cells need to accumulate at the tumour site where they can affect the TME [10]. Activation of the immune system against the tumour, however usually results in an “arms race” with “survival of the fittest” tumour cells enabled by genomic instability and somatic cellular Darwinian evolution and adaptation [146,147,148]. Various mechanisms contribute, including rapid tumour cell replication, mutations that favour tumour growth, and immune evasion by cancer immunoediting [17,145]. This involves antigen loss by immunologic sculpting to eliminate tumour cells with high immunogenicity leaving behind tumour variants with reduced immunogenicity [149] and resistance to immune effector cells. Cancer cells are also able to eliminate non-silent point mutations (that are recognised by T-cells) more frequently than silent point mutations [150]). Other consequences include immunosuppression through lymphocyte apoptosis and activation of negative regulatory pathways as well as induction of tolerance [17,86,134,145]. In addition to the generally immunosuppressive environment associated with cancer, normal T-cells may not efficiently recognize tumours because of downregulation or absence of MHC expression, or weak immunogenicity of tumours since they are not phenotypically foreign [151]. For example, analyses in humans have revealed that the TCRs from T-cells that recognize self-tumour antigens have a substantially lower affinity (approximately 1.5 logs) compared to virus-specific TCRs [152], due to negative selection of lymphocytes expressing high-affinity antigen receptors to normal differentiation antigens during development [17].

Immune suppression due to the metabolic transition that occurs in cancer cells for ATP production, (known as the Warburg effect), from mitochondrial oxidative phosphorylation (OXPHOS) to cytoplasmic aerobic glycolysis (conversion of glucose to pyruvate), is another key mechanism of immune evasion [153,154]. In cancer cells, this glycolysis can occur under aerobic or hypoxic conditions and generates ATP and lactate, with increased acidity in the TME [153,155]. T-cells themselves also normally depend on glucose and glycolysis for their proliferation and effector function, but tumour cells outcompete T-cells in the TME for glucose and other nutrients for their own function [153]. In addition, T-cells within the TME undergo progressive loss of PPAR-gamma coactivator 1-alpha (PGC1α, PPARGC1a gene), which programs mitochondrial biogenesis, resulting in their repression of oxidative metabolism, and effector T-cells with unmet metabolic requirements [156]. Ovarian cancer cells in vitro have been found to impose glucose restriction on T-cells which dampened their function via high expression of microRNAs miR-101 and miR-26a, which limited expression of the methyltransferase EZH2 by the T-cells, and therefore their cytokine expression and survival [157].

In addition to the immunosuppression in the TME by cancer cells’ expression of various cytokines that promote recruitment of immunosuppressive cells, immunosuppression also occurs by their constitutive overexpression of the immunosuppressive intracellular enzyme indoleamine-2,3-dioxygenase 1 (IDO1) in response to interferon [86]. This leads to degradation of the essential amino acid L-tryptophan, thereby decreasing NK cell and CD4+ and CD8+ T-cell proliferation by induction of apoptosis and promotion of T-cell exhaustion, while supporting the activity of Tregs and MSDCs [86,87]. IDO1 protein expression in DCs is upregulated by CTLA-4 [87], and is associated with paclitaxel resistance [158] and poor prognosis of serous ovarian cancer patients [159]

Mutated proteins in cancer cells are normally degraded by the ubiquitin-proteasome pathway and presented by MHC class I molecules to cytotoxic CD8+ cells [160]. Defects in the genes or proteins of the antigen presenting machinery of tumour cells are immune escape mechanisms that disrupt the ability of antigen-specific cytotoxic CD8+ T-cells to recognise and eliminate tumour cells. Such defects have frequently been found in malignancies (including primary and metastatic ovarian cancer lesions) and correlate with tumour grade, stage, disease recurrence, and survival [160]. Alternatively, cross-presentation of antigen by professional APCs including DCs also occurs. In this case, peptides degraded by the lysosomal pathway as a result of endocytosis or autophagy which would normally be presented by class II MHC molecules to CD4+ cells, are instead presented by MHC class I to CD8+ cells [160]. Successful cross-presentation by DCs is critical for induction of a successful antitumor immune response and successful immunotherapy [161]. Defects in trafficking of peptide-MHC class I complexes to the surface of DCs in cancer can occur due to the accumulation of electrophilic oxidatively truncated lipids bound to chaperone heat shock protein 70 [161].

Evasion of immune recognition through downregulating MHC class I expression by tumour cells has the effect of compromising killing of cancer cells by TAA-specific CTLs, but enhances recognition and killing by NK cells which are activated when total levels of MHC I fall below a threshold [17]. Tumours develop resistance to CTL-mediated killing mechanisms by inhibiting the perforin/granzyme pathway through expression of granzyme-specific serine proteases (serpins); and may express decoy receptors, e.g., soluble Fas and decoy receptors 3 and 4 (DcR3, DcR4) for the death receptors Fas and tumour necrosis factor-related apoptosis-inducing ligand (TRAIL) [17]. Upregulation of expression of antiapoptotic and prosurvival molecules such as BCL2, STAT3, cFLIP, and BCLXL by tumour cells may also occur [17].

TAMs in the TME also play a role in immunosuppression, including suppression of T cell activation [162] and responses [163]. Potential mechanisms involve hypoxia and hypoxia inducible factor 1-α (HIF-1α) [164], and B7-H4 expression on TAMs [162]. TAMs expressing B7-H4 inhibit the activation of antigen-specific T- cells in human ovarian cancer and its inhibition restores TAM function to aid tumor regression [162]. Small-molecule inhibitors to CSF1R also have been shown to reduce TAM populations and to significantly enhance responses to chemotherapy. This effect is thought to be at least in part due to the removal of macrophage-mediated immunosuppression [165]. Another mechanism of immune suppression by tumour cells occurs by their secretion of extracellular vesicles (EVs) including exosomes (approximately 30–150 nm), which are internalised by other cells [166]. Tumour exosomes have been found to express a number of immunosuppressive molecules including surface-exposed phosphatidyl serine [167], the death receptor ligands FasL and TRAIL, immune checkpoint inhibitor ligands (e.g., PD-L1), and inhibitory cytokines (e.g., IL-10, TGF-β1) [166]. Tumour exosomes can also specifically bind and sequester therapeutic monoclonal antibodies (mAbs) thereby preventing binding of the mAb to the tumour cells and the expected downstream cytotoxicity by immune effector cells, as seen for HER2+ breast cancer cell lines treated with trastuzumab (Herceptin) [166]. The nucleic acid content (gDNA, mRNA, miRNA) of tumour exosomes can also reprogram gene expression of recipient immune cells [166].

### 5.3. Tolerance

The immune tolerance of cancer has similarities to the interactions between the embryo/fetus and maternal immune system to prevent rejection in pregnancy. Exploitation and upregulation of negative costimulatory signaling pathways such as CLTA-4/B7 and PD-/PD-L1 occurs at the feto-maternal interface of the placenta [168] as well as between tumour/immune cells. In addition, the expression of MUC16 (cancer antigen 125, CA125 [169]) in the endometrium is thought to prevent uterine NK cells attacking the trophoblast [170] and upregulation of MUC16 is typically found in ovarian, pancreatic, and other cancers [171,172,173,174].

### 5.4. The Role of “Classical” Checkpoint Inhibitors

The most important peripheral checkpoint inhibitor pathway exploited by tumour cells within the TME from a clinical perspective to date, is the interaction between the PD-1 receptor on T-cells with its ligands PD-L1 and PD-L2 on tumour cells [175]. Increased activation of the pathway results in increased Treg recruitment and tolerance [175], with reduced effector T-cell proliferation, cytokine production, and survival [176]. PD-1 is highly expressed on TILs [50], particularly CD8+ immune effector cells [177]. PD-L1 (CD274/B7-H1, a transmembrane protein) is an anti-apoptotic factor for tumour cells resulting in resistance to cytolysis by CTLs and drug-induced apoptosis, and engagement of PD-L1 with its receptor PD-1 on T-cells additionally triggers apoptosis of CTLs [86]. An early study of PD-L1 expression in cancer found that high protein expression of PD-L1 on tumour cells from a mixed group of ovarian cancer histological subtypes, was associated with significantly poorer patient prognosis compared to low expression, and was inversely correlated with numbers of intraepithelial CD8+ tumour infiltrating lymphocytes (TILs) [31]. PD-L2 protein expression was not associated with patient outcome [31]. In contrast, a more recent study with larger numbers of histological subtypes of ovarian cancers and different PD-L1 antibodies, found PD-L1 expression mainly on TAMs (CD68+), and was strongly associated with cytolytic and regulatory TIL subsets [178]. Overall, PD-L1 expression resulted in a net positive association with survival for HGSOC, but no prognostic significance was found for the other ovarian cancer subtypes [178].

### 5.5. Protein Glycosylation, Siglecs, and Mucins: Alternative Checkpoint Inhibitors

Aberrant protein glycosylation, particularly O-linked glycosylation, has been noted as an underlying mechanism of all cancer hallmarks [179]. Protein glycosylation is the most common and complex post-translational modification, with over 50% of proteins estimated to be glycosylated, and over 1% human genes contributing to this process [180]. It is involved in many physiological processes, including cell-cell and cell-matrix interactions, barrier function, cellular signaling and differentiation, as well as immune signaling and modulation of immune responses [12,180,181]. Aberrant O-linked protein glycosylation is frequently found on the surface of cancer cells and influences the ECM and cancer cell growth, proliferation, resistance to cell death, capacity for invasion, and is associated with poor prognosis of cancer patients [179,182]. The first step of O-linked glycosylation is the addition of N-acetylgalactosamine (GalNAc) at the hydroxyl group of serine or threonine and forms the Tn-antigen (Thomsen-nouveau antigen, or Tn-epitope) (GalNAcα1-O-Ser/Thr, CD175) [182,183]. This structure can then be extended to form simple or complexed branching saccharide chains and can be frequently sialylated (STn-antigen, CD175s) in cancer [182]. The most common glycosylation alterations in cancer include increased global sialylation, fucosylation, *O*-glycan truncation, and *N*- and *O*- glycan branching [184]. Cell surface sialosides play a major role in immune modulation that is exploited by tumours to evade destruction by the innate and adaptive immune system [58]. Sialic acids are found mostly at the tip of glycans, including those present on mucin proteins, where they are strategically positioned to influence cell-cell interactions [56]. In contrast to PAMPs and DAMPs, glycans such as sialic acids serve as a self-associated molecular pattern (SAMP) recognised by innate immune inhibitory receptors to maintain a non-activated state of the immune system and prevent autoimmune responses [185,186]. Generally, aberrant tumour cell glycosylation results in new connections with immune cells that actively suppress anti-tumour immunity [185]. It has been suggested that since tumour-specific glycosylation patterns determine the immune inhibitory nature of the tumour, these glycan-lectin interactions should be considered as novel immune checkpoints that can be targeted by immunotherapy [185]. Hypersialylation alters immune cell responses to cancer by modulating immune cells involved in inflammatory responses, including via Siglecs present on NK cells and correlates with poor prognosis and decreased tumour immunogenicity [58]. Hypersialylation can recruit NK cell associated siglecs to an immunological synapse that mediates suppression of NK activating signals via ITIM domains present on most siglecs [58]. In addition, cancer-associated structural alterations regarding the type and composition of sialic acids are found on different glycoconjugates, including mucins [187]. Xenosialylation, with incorporation of the nonhuman sialic acid *N*-glycol-neuraminic acid (Neu5Gc) from dietary sources into human glycans are associated with cancer progression [188], and increased levels have been observed in ovarian cancer [189]. Aberrant overexpression and glycosylation of transmembrane epithelial mucins occurs in various cancers and contribute to disease progression and metastasis [190]. MUC1 undergoes various glyco-modifications during ovarian cancer progression [191], and is also overexpressed in other adenocarcinomas and haematological malignancies [86]. MUC16 is overexpressed primarily in ovarian cancer [86] with soluble, tumour-shed antigens or membrane bound forms able to suppress humoral-based immunity, particularly antibody-dependent cell-mediated cytotoxicity (ADCC) [192,193]. These mucins are ligands for Siglecs, inhibitory receptors on innate and adaptive immune cells that may contribute to dampening of the immune response against tumours. Siglec-7 is highly expressed on NK cells and engagement with Siglec-7 ligands on cancer cells inhibits NK cell-mediated killing [56]. Siglec-9 is expressed on the surface of B-cells, granulocytes and NK cells, and its interaction with membrane-bound MUC16 may pose a barrier to NK cell interaction with cancer cells, while interaction with soluble MUC16 downregulates the activating Fc-γ receptor CD16 on NK cells [194]. Siglec-9 is also present on macrophages in the TME [56], and tumour-infiltrating T-cells with co-expression of PD-1 in ovarian cancer patients [186]. Truncated, short sialylated O-glycan forms present on cancer cells enable their direct interaction with Siglec-9 on myeloid cells and promote myeloid differentiation into immunosuppressive TAMs [195]. Siglec-9 is rarely expressed on human T-cells, however, it has been recently reported that a subset of intratumoral (though not peripheral) melanoma CD8+ T-cells, express Sigelc-9. It was demonstrated that these cells were functionally inhibited in the presence of Siglec-9 ligands or engagement with Siglec-9 antibodies [196]. It was proposed that targeting the tumour-restricted glycosylation-dependent Siglec-9 axis may activate these Siglec-9+ CD8+ T-cells with confinement of their actions to the TME, and prevent uncontrolled systemic T-cell activation and associated toxicities [196]. Subsets of Siglec-9+ expressing CD8+ and CD4+ TILs have also been found in patients with epithelial ovarian cancer [186].

## 6. Agents and Strategies for Cancer Immunotherapy

### 6.1. Conventional Cancer Therapy and Immunotherapies

Conventional cancer therapies such as debulking surgery, molecularly targeted therapies, chemotherapy, and radiotherapy for metastatic lesions are aimed at directly targeting the tumours themselves, but nevertheless also affect the immune system. Standard cytotoxic chemotherapy initially decreases the patient’s own immune competent cells including T-cells which then recover [145] and can recognise cancer cells due to antigen recognition prompted by cancer cell apoptosis and their release of DAMPs [197]. Repeated cycles of chemotherapy, however, may result in lymphopenia and loss of cell-mediated immune function [145].

Strategies for cancer immunotherapy may aim to either stimulate the immune system with immune effectors or to modulate the immune system to inhibit the inhibitors of the immune system [48]. Immune system stimulation may engage the innate immune system, e.g., NK cells, and/or the adaptive immune system by enhancing T-cell activation with various interleukins, and/or costimulatory signals to increase their proliferative activity and longevity, thereby enhancing the strength and duration of the immune response. In contrast, immune system inhibition may aim to block immune checkpoint inhibition, immunosuppression of the TME, or tolerance to cancer by targeting Tregs [10]. Immunotherapies have also been classified as passive or active. Passive immunotherapies deliver generic lymphocytes, antibodies, or other components of the immune system; while active immunotherapies rely on agents to specifically stimulate the patient’s own immune responses, such as the production of antibodies or lymphocytes against tumour cells [198]. Agents used for the various strategies have included bacteria, viruses, mAbs, therapeutic vaccines, or adoptive transfer of cells (TILs, T-cells, DCs, NK cells), with or without TCRs, antigen specific chimeric antigen receptors (CARs), and immunostimulatory cytokines. Table 3 summarises specific immunotherapeutic agents used clinically for various cancers, or being assessed in clinical trials for ovarian cancer. It should be noted that despite numerous relevant ovarian cancer clinical trials, less than 10% of registered and 15% of registered and completed trials at the US National Library of Medicine ClinicalTrials.gov website, have results published in journals indexed on Medline and PubMed or posted with ClinicalTrials.gov [199].

For detailed reviews of antibody-based immunotherapies for ovarian cancer see Tse et al., 2014 [200] and Drerup et al., 2015 [201]. For more recent reviews of immune therapies for ovarian cancer with engineered T-cells, TCRs, and CAR-T cells see Marth et al., 2019 [8]; Fan et al., 2018 [53]; Rodriguez et al., 2018 [73]; Zhu et al., 2017 [202]; Rodriguez-Garcia et al., 2017 [203]; Gaillard et al., 2016 [52]; Alipour et al., 2016 [204]. These include summaries of recruiting and ongoing clinical trials targeting immune checkpoint inhibitors or various antigens including, NY-ESO-1, HER2, FR-alpha, MSLN, MUC16 (CA125), EGFR, CD133, CEA, NKG2D, MAGE-A4, WT-1, and p53.

### 6.2. Bacteria-Mediated Tumour Therapy

The earliest attempts to exploit the immune system to fight cancer utilised activation of the innate immune system by pathogens. In historical times from ancient Egypt to 19th century Europe, it was noticed that tumorous growths could sometimes regress in patients with simultaneous infection and fever. In 1868, Wilhelm Busch was the first to infect a patient with inoperable sarcoma intentionally, with *Streptococcus pyogenes* bacteria from the skin disease erysipelas, resulting in high fever. (The tumour shrank, but the patient died days later). In 1891, William Coley began a 43-year project of treatment of inoperable cancer patients with heat-inactivated bacteria known as “Coley’s toxins”. There was some success, but a balance between the toxic effects of the bacteria and therapeutic efficacy was difficult to achieve [252]. The strategy lost favour with the advent of radiotherapy and chemotherapy [253], until the 1960s and ‘70s with the use of BCG bacteria (related to tuberculosis-causing bacteria) for successful treatment of bladder cancer [254,255]. Preclinical studies with mice have shown that some bacteria, including *Salmonella*, *Escherichia*, *Clostridium* and *Bifidobacterium* specifically accumulate at the site of tumours in response to chemotactic signals from the TME [256], and may proliferate to numbers that far exceed the number administered due to the distinct metabolic and immunosuppressive nature of the TME [257]. Intracellular multiplication of *Salmonella* can lead to bursting of invaded tumour cells, or may instead induce autophagy or apoptosis. Lipopolysaccharide (LPS) present on the outer membrane of gram-negative bacteria including *Salmonella*, is a potent stimulator of TNF expression by macrophages; while flagellin, a protein subunit present in the bacterial flagellum improves the CD8+ T-cell-dependent antitumour response through activation of toll-like receptor 5 (TLR5) by suppressing tumour cell proliferation and induction and activation of NK cells. These abilities are being exploited in clinical trials with live attenuated and engineered tumour-targeting bacteria, e.g., with genetically engineered *Salmonella* as a vector to stimulate the immune system in an optimal way to achieve tumour toxicity. However, early human trial results have shown inferior tumour colonisation and therapeutic effects compared to that seen in preclinical models [257].

The gut microbiota have been shown to modulate the immune system. Recent studies have revealed interaction between the gut microbiome and immune checkpoint inhibitors (ipilimumab) and PD-1 blockers (see below) which influences therapeutic efficacy [255,258]. Melanoma patients that responded well to anti-PD-1 therapy had diverse bacteria with abundant *Faecalibacterium*, *Bifidobacterium longum*, and *Bacteroidales*, while the presence of *Ruminococcus obeum* and *Roseburia intestinalis* was associated with non-responsiveness. Antibiotic exposure during treatment was also associated with a negative response. Specific bacterial species, however, may be optimal for different cancer types [259].

### 6.3. Oncolytic Viruses

Similar to bacteria-mediated cancer therapy, oncolytic viruses are at the junction of biological- and immunotherapy, and are genetically engineered to lack virulence against normal cells. Due to the interaction of viral surface structures and host surface receptors (tropism), oncolytic viruses preferentially infect, proliferate within, and kill cancerous cells by lysis or induction of apoptosis, with subsequent induction of systemic antitumour immunity. They also have the advantage of targeting cancer stem cells and avoiding drug-resistance mechanisms of cancer cells [260]. Moreover, it is apparent that cancer cells have lost many of their normal anti-viral cellular defences in order to amplify their growth potential [254]. In addition, oncolytic viruses can be engineered strategically in various ways. For example, to 1) engage costimulatory molecules, e.g., with expression of CD40L/CD154; 2) enhance cross-presentation of tumour antigens e.g., with heat shock proteins; 3) enhance APC function, e.g., with agonists of TLRs; 4) reduce immune suppression in the TME [261]. Several viruses, including adenovirus, vaccinia virus, and the measles virus have been investigated in clinical trials for cancer treatment after noticing that viral infections can benefit cancer patients [262].

T-VEC (ImlygicTM, Talimogene laherparepvec) is an oncolytic herpes virus modified to express GM-CSF (with the aim to stimulate proliferation of immune cells and differentiation and maturation of DCs [261]). It was the first oncolytic virus approved for cancer therapy by the FDA (in 2015) to treat advanced melanoma by direct intratumoral injection [263] after a phase III clinical trial showed a significantly increased durable response rate (≥6 months) compared to GMCSF treatment alone, though with an overall survival rate that failed to reach statistical significance (*p* = 0.051) [264]. A combination of T-VEC with mAbs against the checkpoint inhibitors CTLA-4 and/or PD-1, however, may improve survival [265,266]. The oncolytic measles virus (MV-NIS), which uses the surface receptor CD46 for entry into cells is being trialled to treat drug resistant ovarian cancer in which CD46 is overexpressed [267,268]. Other clinical trials for ovarian cancer employ oncolytic viruses from the vaccinia, adenovirus, and reovirus families [260].

### 6.4. Cancer Vaccines

In addition to the use of bacteria and oncolytic viruses, which effectively behave as in situ vaccines and stimulate the immune system without the need for isolation and identification of antigens [261], a variety of therapeutic cancer vaccine platforms have been developed and tested. A therapeutic vaccine should be able to induce cell mediated immunity in which immune cells are activated to recognise and destroy their cellular targets in affected tissue [269]. Cancer vaccines include: (1) whole tumour cell vaccines, based on the administration of cancer cell lysates; (2) peptide-based vaccines, based on the direct delivery of recombinant proteins or epitopes in combination with immunological adjuvants; (3) dendritic cell (DC)-based vaccines, most often based on the isolation of patient-derived DCs, matured ex vivo in the presence of TAAs and their re-infusion; (4) RNA-based vaccines, based on the direct delivery of RNA molecules extracted from malignant cells or specifically encoding a single TAA; (5) DNA-based vaccines, based on the administration of naked plasmids or vectored TAA-coding plasmids under the control of a strong mammalian or viral promoter [270]. Naked DNA vaccines rely on the uptake of the vaccine by cells (mostly myocytes, resident DCs and monocytes) with subsequent antigen expression, processing and exposure on MHC Class I molecules, potentially resulting in CD8+ T-cell priming and long-term immunity [270].

A number of ovarian cancer trials have been conducted in patients using vaccinations with ESO 157-170, a short peptide of the CTA NY-ESO-1 [271], and heterologous prime-boost vaccinations [6], i.e., multiple vaccinations with different delivery vectors encoding the same recombinant antigen [272]. Generally, therapeutic cancer vaccines are associated with minimal side effects, but they have shown consistently low efficacy [269].

A promising recent pre-clinical study has found that administration of a vaccine without adjuvant against seemingly irrelevant viruses, could be an effective cancer immunotherapy by converting immunologically cold tumours into hot tumours [273]. A seasonal influenza vaccine administered intratumorally within the TME stimulated systemic CD8+ T-cell-mediated immunity and infiltration of CD8+ T-cells into the tumours, while decreasing intratumoral Bregs. At the same time, such treatment sensitised tumours to immune checkpoint blockade (ICB) immunotherapy [273].

### 6.5. Monoclonal Antibodies (mAbs), Short Chain Variable Fragments (ScFvs) and Bispecific T-Cell Engagers (BiTES)

#### 6.5.1. Monoclonal Antibodies (mAbs)

**Figure 2:**
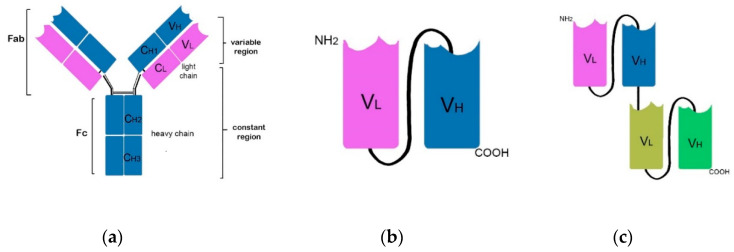
General structures of some common cancer immunotherapeutic agents or components. (**a**) IgG mAb. Fab = antigen binding fragment, Fc = complement and Fc receptor binding fragment. (**b**) Single chain variable fragment (scFv) structure, derived from the heavy and light chains of the variable antigen binding domain of a mAb. (The V_L_ and V_H_ units may be engineered in either order). (**c**) Tandem scFv, bispecific T-cell engager (BiTE) structure. C = constant region, V = variable region, H = heavy chain, L = light chain.

Introduction of mAbs to characterise cell surface proteins of cancerous cells and altered antigenicity of stroma and ECs in the TME (i.e., TAAs) have led to the development of a number of mAbs for cancer immunotherapy [274]. mAbs function as anticancer agents in two main ways. The first is direct action by the antibody by targeting and surface antigen binding via their antigen binding fragment (Fab) domain, which usually blocks (antagonises or neutralises) the function of their target, or may agonise and stimulate their target. Examples of these include trastuzumab and bevacizumab which block HER2 and VEGF, respectively (see below). The second mode of action stimulates the innate immune system via immune-mediated cell killing mechanisms and includes ADCC that leads to phagocytosis, and complement-mediated cytotoxicity (CDC) that induces lysis of the target cell [274,275]. Both ADCC and CDC occurs via the mAb Fc domain interaction with Fc-γ receptors present on immune cells [274]. Direct mAb antigen targeting can modulate tumour cell signaling pathways, or interfere with tumour-stroma interactions [276]. Tumour cell lysis by ADCC or CDC can enhance uptake and cross-presentation of antigens by DCs leading to stimulation of adaptive immune responses [10]. See Figure 2a for mAb structure with Fab and Fc domains. The efficacy and clinical success of mAbs depends not just on antigen recognition and binding, but also on the presence of effector immune cells with antibody-dependent cytotoxicity activity in the tumour infiltrate, including NK cells [151].

In contrast to most mAbs which are antagonistic for their targets, antibodies targeting different co-stimulatory members of the TNFR superfamily (e.g., CD27, CD134, CD137, CD357), are agonists which induce or facilitate receptor-mediated downstream signaling. These have been developed for cancer immunotherapy with some success in animal models and are in early phase clinical trials, usually in conjunction with other immunotherapies [27]. Varlilumab, an agonist antibody targeting CD27 is in clinical development for the treatment of haematological and solid malignancies [277], including ovarian cancer [227].

#### 6.5.2. Single Chain Variable Fragments (scFvs) and Bispecific T-Cell Engagers (BiTES)

Catumaxomab, a hybrid of two hybridomas targeting different antigens, CD3 and epithelial cell adhesion molecule (EpCAM), is a bispecific antibody with trifunctional activity. This trifunctional ability of catumaxomab allows it to bind FcγR on NK cells, DCs or macrophages via its Fc domain with simultaneous binding to 3 different cell types, i.e., a T-cell, tumour cell, and accessory cell, and provides secondary T-cell stimulation [278,279]. Concern regarding undesirable effects of Fc receptor interactions, however, drove development of T-cell targeting bispecific antibodies without Fc receptors, i.e., bispecific T-cell engagers (BiTES) [279]. BiTES are a subclass of bispecific antibodies with two different antigen binding sites, one on the tumour target and one on the T cell, and are tandem single chain variable fragments (scFvs). An scFv is a fusion protein from the variable antigen binding domains of the heavy and light chains from an antibody (Figure 2a), often a mouse mAb. Each scFv of a BiTE has a unique antigen specificity that targets a tumour-specific antigen on one Fab arm and the TCR complex (often CD3ε) on the other, separated by a linker [279,280]. See Figure 2b,c for the generalised structures of an scFv and BiTE. Linking a cytotoxic T-cell to a tumour cell in this way enables cytotoxic activity against the tumour despite the absence of co-stimulation [281]. An example of a BiTE in clinical trials is blinatumomab, targeting CD19 for B-cell malignancies [279]; others are MT110 (solitomab) targeting EpCAM [279,282] and mouse- and human-specific B7-H6 BiTEs [283]. This latter TAA is a specific ligand for NK cells’ activating receptor NKp30, and these BiTEs have been found to decrease tumour burden in a mouse model of ovarian cancer [283].

As mentioned above, catumaxomab, which targets EpCAM andCD3, was approved for the intraperitoneal use in ovarian cancer patients and others with malignant ascites and EpCAM+ tumours in Europe in 2009 [278,279]. A clinical trial found that catumaxomab significantly improved malignant ascites in patients with epithelial ovarian cancer and patients with non-ovarian cancer and significantly increased drainage-free survival and time to next paracentesis. However, there was no significant increase in overall survival in the ovarian cancer group [284].

### 6.6. Target Choice for Immunotherapy

In contrast to haematological cancers, which present with CD19/CD20 as a specific B-cell targets for immunotherapy with demonstrated effectiveness, solid tumours including malignant epithelial cells are diverse and their target antigens more heterogeneous, resulting in not just poor specificity but also poor efficacy [151]. Consequently, the immunotherapeutic strategies employed against solid cancers are more diverse. In addition to target antigen choice, its immunogenicity needs to be considered, which depends on its level of expression, how it’s processed and presented, and how well it is recognised by T-cells. Induction of an immune response to irrelevant antigens could misguide the immune system and lead to failure to attack tumour cells. It has been proposed that solid tumours will be defined by their immunogenicity, i.e., ability to induce or enhance the activity of tumour-specific T-cells, and thereby determine their method of immunotherapy [285].

A diverse range of mAbs and targets have been employed for solid cancer immunotherapy. These include tumour promoting molecules such as VEGF to provide anti-angiogenic therapy (e.g., bevacizumab), IL-6, and MIF (macrophage migration-inhibitory factor), immune checkpoint inhibitors (e.g., PD-1 and CTLA-4), as well as TAAs, e.g., HER2 [286].

#### 6.6.1. Anti-Angiogenic Therapy

VEGF, an important mediator of angiogenesis, is overexpressed in most human malignancies [275] including HGSOC and secreted into malignant ascites, and is associated with reduced survival in OC patients [72]. It is produced by tumour cells as well as non-cancerous cells of the TME including CAFs, adipocytes, and leukocytes [72]. Bevacizumab was first approved in 2004 by the FDA for treatment of metastatic colorectal cancer. It was approved by the FDA for ovarian cancer in 2014, specifically for treatment of advanced recurrent platinum-resistant ovarian cancer in combination with chemotherapy [9]. A phase III randomised trial of patients with platinum resistant ovarian cancer compared chemotherapy with or without bevacizumab (AURELIA, NCT00976911). Increased overall survival (*p* = 0.001) was found in patients receiving bevacizumab compared to those receiving chemotherapy alone (*n* = 110) [287]. However, a phase III randomised trial of bevacizumab with chemotherapy in women with newly diagnosed ovarian, fallopian tube, or primary peritoneal cancer (NCT00262847) found no survival differences for patients receiving bevacizumab compared to those receiving chemotherapy alone [288]. It has been suggested that the relative lack of efficacy of anti-angiogenic treatment in ovarian cancer may be due to an increase in vasculogenic mimicry after bevacizumab treatment [129], although it is likely that there are multiple other pathways for resistance to anti-angiogenic therapy [289].

#### 6.6.2. TAAs

Many targets for solid tumours in clinical trials for ovarian cancer have relied on well-established tumour-associated antigens, e.g., HER2, WT1, NY-ESO-1, and p53, but these are not frequently found to be presented on MHC molecules on ovarian cancer cells. Comparative profiling of MHC-presented antigens from epithelial ovarian tumours vs. benign tissues instead found numerous immunogenic targets including MUC16, mesothelin (MSLN, a ligand of MUC16), LGALS1, IDO1, and KLK10 [30]. In a variety of cancers, including ovarian cancer, MAGEA proteins (part of a large family of melanoma antigen genes, (MAGEs)—also a subgroup of CTA antigens) are found more frequently and with higher expression levels than NY-ESO-1 and are therefore thought to represent more promising and potent targets for immunotherapy [138]. The 2017 Schuster study also showed that NY-ESO-1 peptide antigen was not presented by MHC-I or MHC-II molecules in any of 42 epithelial ovarian cancer samples, although peptides from various MAGEA family members were presented [30].

#### 6.6.3. Immune Checkpoint Modulation

The immune checkpoint interactions between TCRs and APCs that determine whether the immune system is stimulated or inhibited, have provided effective targets for immunotherapeutic approaches to cancer treatment. Blocking inhibitory immune checkpoint interactions between T-cells and tumour cells responsible for tolerance, i.e., blocking immune suppressor signals such as CTLA-4 and PD-1, has been a powerful strategy for cancer immunotherapy [290]. CTLA-4 (CD152) is a T-cell receptor with an inhibitory function that blocks the activating signal 2 by binding the same APC CD80 or CD86 molecules with higher affinity than CD28 [48,49]. CTLA-4 is predominantly expressed on CD4+ and not CD8+ CTLs [50]. Neither CD80 nor CD86 are expressed on non-haematological tumour cells, so the main effects of CTLA-4 are thought to occur within the secondary lymphoid organs [50]. CTLA-4 function results in dampening activation of effector CD8+ T-cells as well as driving the suppressive function of CD4 Tregs to maintain immune tolerance [50]. Tregs have strong expression levels of CTLA-4 [51].

Preclinical models in the 1980s and 1990s with monoclonal antibodies to CTLA-4, and finally in clinical trials with ipilimumab demonstrated significantly increased survival for some patients with advanced melanoma [48,290]. Serious side-effects, however, were also seen in some patients, particularly inflammation [290]. Such immune-related toxicities are thought to be due to indiscriminate activation of auto-reactive T-cells, which are usually supressed via these molecules [8]. Only 2 patients out of 40 with recurrent platinum sensitive ovarian cancer completed a phase 2 clinical trial with Ipilimumab monotherapy (NCT01611558), with failure to complete mostly due to study drug toxicity or disease progression [291]. Tremelimumab is another anti-CTLA-4 mAb in clinical development and trialled in ovarian cancer patients in conjunction with poly (ADP-ribose) polymerase (PARP) inhibitors [175].

PD-1 is found on T-cells activated by TCR engagement with antigen in response to phosphorylation of ITAMS present within CD3 (Figure 1), which leads to interferon production by the T-cells. Interferon production normally promotes the immune response and attracts NK cells and macrophages, but it also leads to immunosuppressive factor production in order to limit the immune and inflammatory response [150]. Cancer cells respond to interferon by production of a PD-1 ligand, transmembrane protein (PD-L1, CD274/B7-H1). PD-L1 and PD-L2 are also expressed in other cellular components of the TME including macrophages (mostly M2), myeloid DCs, MDSCs, stromal fibroblasts, and endothelial cells [292]. Engagement of PD-1 with PD-L1 results in negative regulation of T-cell mediated immune responses, including inhibition of T-cell proliferation, survival, cytokine production, and other effector functions [293]. Upregulation of PD-L1 within the TME is the basis for PD-1/PD-L1 blockade therapy, however this upregulation of expression is limited to the tumour-stroma interface [294]. Antibodies to PD-1 (nivolumab and pembrolizumab) and PD-L1 (durvalumab, avelumab, atezolizumab) have been developed for cancer immunotherapy, and have been tested as monotherapies and combined in clinical trials with ipilimumab [48,295], including for epithelial ovarian cancer [8]. Although clinical benefits and durable responses have been seen in multiple tumour types, primary resistance or acquired resistance to PD-1/PD-L1 blockade is a major problem [294]. Primary resistance may be due to a relative lack of tumour PD-L1 expression, with data for various cancers showing that patients with tumour biopsies with PD-L1 positivity by immunohistochemistry have higher response rates than those with PD-L1 negative disease [294]. Another contributing factor to primary resistance is insufficient pre-existing CD8+ TILs at the invasive tumour margin [294]. Based mainly on work on melanomas, it has been proposed that the TME of tumours can be classified based on their presence or absence of TILs and expression of PD-L1, with a view to rational design and prediction of response to PD-1/PD-L1 ICB immunotherapy for cancer patients [177]. It is likely that a similar rationale will be beneficial to understanding and predicting the response to checkpoint inhibitors in the treatment of ovarian cancer [52]. Four main types of tumour TME were observed, with the tumours most likely to respond being positive for both TILs and PD-L1, and classified as Type I [177]. A similar type I TME has been found to be the most common type found in HGSOC, and more common in this histologic subtype compared to other ovarian cancers [52,178]. The other three TME types are: type II, negative for both PD-L1 and TILs; type III, PD-L1+ TIL-; and type, IV PD-L1- TIL+. A type IV TME pattern was the most prevalent type for low-grade serous, mucinous, endometrioid, and clear cell ovarian cancers [52,178]. However, PD-L1 expression and TIL infiltration have not yet been shown to be useful for ovarian cancer patient selection [296]. PD-L1 expression has only been observed in 10–33% of epithelial ovarian cancers [297] and its role in ovarian cancer prognosis is still inconclusive [178,298]. Many issues have recently been highlighted for using PD-1 expression to predict response to immune checkpoint inhibitors, including: intratumoral heterogeneity; testing on archival tissues may not reflect current disease; use of different antibodies and scoring methods; and determining the optimal cut point [299]. There are similar issues in assessing TIL infiltration in ovarian cancer tissues [299]. Nevertheless, a greater understanding of the TME together with improved genetic classification and assessment of somatic mutations and mutational load, will aid in patient selection and predicting response to treatment [299].

In ovarian cancer, objective response rates to checkpoint inhibitors as single agents in clinical trials was estimated to be only 10–15% [52,300], or 6–22% [8,301]. A recent retrospective analysis of epithelial ovarian cancer patients receiving ICB immunotherapy of targets including PD-1, PD-L1, CTLA-4, and LAG-3 alone or in combination, found that over 50% of patients suffer disease progression requiring early discontinuation of therapy [302]. The median progression-free survival reported was very short, and typically coincided with the first protocol-defined scan, leading to treatment discontinuation in the majority of patients [302]. Interestingly, one study found a strong correlation between the objective response rate to anti–PD-1 or anti–PDL1 therapy in 27 tumor types, including ovarian cancer, and overall tumour mutational burden [303].

The mechanism of action of immune checkpoint blockers to impair T-cell inhibition and overcome T-cell exhaustion, and anergy leads to a loss of self-tolerance and related toxicities, which are more common and severe with anti-CTLA-4 therapy than anti-PD-1/PD-L1 [52]. These side effects or immune related adverse events (irAEs), are often driven by off-tumour inflammation and autoimmunity [304]. Such side effects vary in their timing after treatment, and may be dermatologic, rheumatologic, endocrine, or gastrointestinal, and are mostly reversible although can be life-threatening [52]. The incidence of high-grade irAEs is also higher with combination-immunotherapies compared to single agent immunotherapies [305]. A recent clinical trial with advanced ovarian cancer patients and nivolumab in combination with varlilumab, found one of eight patients experienced dose limiting liver and renal toxicity, though overall treatment was correlated with increases in CD8+ T-cell infiltration and decreases in circulating Tregs [228]. Despite the low response rates seen with checkpoint inhibitors as monotherapies in ovarian cancer, the majority of clinical trials involving thousands of patients for immunotherapy in ovarian cancer in the next year will include checkpoint inhibitors [8]. It is thought that applying such agents early in the disease may reverse T-cell exhaustion caused by long-term exposure to TAAs and rounds of chemotherapy [8].

Acquired resistance to PD-1/PD-L1 ICB may occur due to loss of mutation-associated antigens and/or acquisition of other mutations, e.g., JAK1/JAK2 (normally responsible for IFN-related signaling), or beta-2-microglobulin function (which is normally required for cell surface expression of MHC-I) [306]. A mouse model of ovarian cancer has shown that in vivo treatment with single agent antibodies against PD-1, CTLA-4, or LAG-3 leads to compensatory upregulation of other checkpoint inhibitor pathways in tumour-associated lymphocytes. The upregulation could be overcome by dual blockade with antibodies against PD-1 and CTLA-4 [113].

LAG-3 (CD223) is another immune checkpoint inhibitor targeted with mAbs in the clinic. Persistent antigen exposure in the TME results in sustained T-cell LAG-3 expression, which acts synergistically with PD-1 to promote T-cell exhaustion with reduced proliferation and cytokine production [307]. Relatlimab, an anti-LAG-3 mAb, is being trialled with and without nivolumab for various solid cancers including ovarian cancer (NCT01968109). The trial began in 2013 and is still recruiting [308].

#### 6.6.4. Combination Treatments: PARP Inhibitors and Immune Checkpoint Inhibition

It is also anticipated that a combination of checkpoint inhibitors with antiangiogenic and/or PARP inhibitors may overcome negative influences of the TME and improve ovarian cancer patient outcomes [8,52]. Although not immunotherapy, PARP inhibitors have become important for treatment of ovarian cancer. There are at least 18 PARP family member proteins that normally enable a number of cellular DNA damage repair processes [309]. When inhibited in cells with already compromised alternative DNA repair mechanisms (particularly homologous recombination) as found in HGSOC, the accumulated DNA damage leads to cell death [309,310]. PARP inhibitors were originally FDA-approved for treatment of BRCA-mutated ovarian cancer, however, the latest to be FDA-approved in 2017, niraparib, is also applicable for non-BRCA-mutated ovarian cancer [310]. These agents are being increasingly tested in conjunction with checkpoint inhibitors [296].

### 6.7. Antibody-Drug Conjugates/Immunotoxin Fusion Proteins

Immunoconjugates combine the specificity of mAbs with the potency of cytotoxins. Antibody–drug conjugates/immunotoxin fusion proteins target TAAs expressed on the cell surface via a mAb, antibody fragment, or ligand directed against a cell surface receptor, conjugated via a linker to a toxin which may be bacterial- or plant-derived [311,312]. They are inert in the circulation and their function relies on antigen binding followed by internalisation into the cell with delivery of the toxic payload and subsequent apoptosis [313]. They also provide an effective approach to overcoming immune tolerance [10]. The design of antibody drug conjugates has evolved, with improvements to the 3 major components aimed at effective killing of target cancer cells with high specificity, reduced immunogenicity to overcome resistance, and control bystander killing of cells [311,314]. Various antibody drug conjugates have been FDA approved for use in haematological cancers and advanced stage breast cancer, and several candidates have been used in clinical trials that include patients with epithelial ovarian cancer [311,315]. Side effects of antibody–drug conjugates are generally off-target toxicities that relate to the cytotoxin component used, which are often analogs of auristatins (e.g., MMAE and MMAF) or maytansinoids (e.g., DM1 and DM4) [311]. Adverse events have included hematologic and hepatic toxicity, neuropathy, gastroinstinal, and ocular disturbances [311]. Ontak (denileukin diftitox) is an example of a fusion protein consisting of an enzymatically active domain of diphtheria toxin and the full length sequence of IL-2, which targets cancers expressing the IL-2 receptor [316]. It was tested in a phase II trial of advanced-stage epithelial ovarian cancer in order to deplete Tregs (NCT00880360) [317]. Although functional Tregs from blood and the TME were significantly depleted, there was no significant clinical efficacy [318]. Examples of other antibody–drug conjugates targeting alternative antigens including folate receptor alpha, mesothelin, and MUC16, are included in relevant sections below.

### 6.8. Adoptive Cell Therapy/Transfer (ACT)

#### 6.8.1. Adoptive Cell Therapy with TILs

Adoptive cell therapy/transfer (ACT) involves the intravenous infusion of natural or engineered autologous or allogeneic immune effector cells following ex vivo expansion and activation. Currently, ACT can be classified according to its mechanism of action: namely ACT with tumour-infiltrating lymphocytes (TILs); ACT using T-cell receptor (TCR) gene therapy; and ACT with chimeric antigen receptor (CAR) modified T-cells [319]. Most cancer cell therapies have employed transfusion of autologous T-cells into patients, including CD8+ CTLs alone or combined with CD4+ helper (Th) cells, although NK or cytokine-induced killer cells and macrophages have also been used [320,321].

Early strategies for ACT in the late 1980’s to early 1990’s using cell based therapies for solid tumours in humans focussed first on autologous TILs extracted from metastatic melanoma samples, expanded in vitro with IL-2 to produce an early form of NK cell therapy [322]. Those cells known at the time as “lymphokine-activated killer”, LAK cells, were then transferred back into the same patients, achieving regression in 60% of patients [323]. Limitations of this therapy included inconsistent results with side effects of the IL-2 treatment and co-expansion of Tregs [322]. Generally, a limitation of the clinical success of ACT has been the lack of persistence of transferred cells in vivo. Pre-treating patients with chemotherapy and radiation with resulting lymphodepletion however, increases the persistence and functionality of the transferred cells [324]. A number of small trials with ACT of autologous TILs in patients with ovarian cancer have been conducted in the past, however, although safe, response rates were variable and not statistically significant [325]. Improvements to the ACT methodology with solid tumours have been made, such as pre-conditioning with lymphodepletion and T-cell maturation and maintenance with IL-2 infusions, and new ovarian cancer clinical trials utilising the principles learnt are underway [325], though results are not yet available.

A more selective approach to expand ex vivo only the cells able to recognise antigens relevant to the cancer being targeted, e.g., HPV-associated proteins E6 and E7 in patients with cervical cancer, may be beneficial. However, in a clinical trial testing this approach, only two of nine patients showed a complete response, and further characterisation of the T-cell reactivity from these two patients showed that their TILs actually recognised mutated neoantigens and germline antigens not previously recognised by the patients’ immune system, rather than the selected HPV antigens [326]. This study shows that in order for T-cell based cancer therapy to be more successful, a more effective patient-specific approach to find the most relevant TAAs in an individual’s tumour need to be developed and targeted prior to T-cell selection [327].

#### 6.8.2. ACT with Engineered T-Cells

Not all cancers contain sufficient TILs, and the technical difficulty associated with identifying, isolating and expanding tumour-reactive lymphocytes has encouraged development of engineered T-cells [25]. Newer versions of ACT rely on retroviral engineered T-cells, with either transgenic TCRs or CAR modified T-cells (CAR-T), for specific epitope recognition [327].

Targeted antigens ideally need to be selectively expressed by the tumour without evoking an auto-immune response to the normal “self” [135]. Early trials with engineered TCRs for ACT recognised the melanoma-associated antigen MART-1 (MLANA gene), however they were not successful since they were either ineffective or resulted in severe toxicity caused by cytokine release and macrophage activation [327]. The cancer testis antigen MAGE-A3 has also been used, but one TCR targeting MAGE-A3 resulted in severe neurotoxicity due to cross-reactivity with a similar protein expressed in normal brain, while a different TCR targeting MAGE-A3 caused cardiotoxicity due to cross-reactivity to a different protein [327]. Engineered TCRs that recognise NY-ESO-1 have had some clinical success against melanoma, synovial cell sarcoma, and multiple myeloma, and trials that include patients with ovarian cancer patients are underway [327].

Similarly to ACT with TILs, trafficking of the genetically modified ex vivo expanded T-cells to tumour sites, T-cell persistence and survival are also important factors contributing to the success of these immunotherapies. Greater T-cell persistence and survival is observed in those patients with a complete response to immunotherapy [328].

CARs are synthetic engineered molecules designed for immunotherapy, whose structure is based on the combination of the antigen-binding capacity of monoclonal antibodies together with the intracellular signal transduction domains required to promote activation, lytic capacity and self-renewal of T-cells without TCR α or β chains. CARs overcome several limitations of engineered TCRs. Unlike engineered (or wild-type) TCRs that require MHC intermediaries on APCs for the activation of the T-cells, MHC expressing APCs are not required for CARs and therefore bypass some of the mechanisms by which cancer cells avoid recognition [327,329]. For example, they can recognise tumour cells that have lost their MHC expression or downregulated their proteasomal antigen processing. Furthermore, they can be used in any patient regardless of their MHC-type [324,330]. In addition, because antibody binding domains can recognise native proteins via discontinuous epitopes, the repertoire of specificity for the CAR-T is potentially broader than TCRs, which are restricted to peptides processed for presentation on MHC.

**Figure 3:**
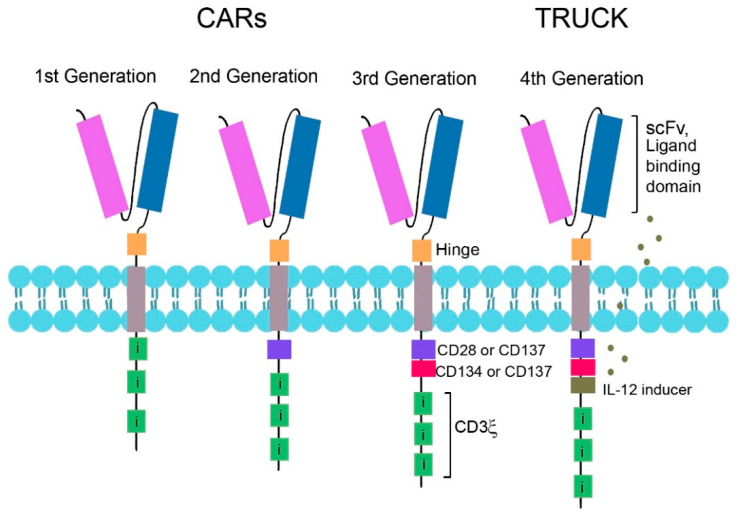
Chimeric antigen receptor (CAR) designs. Target binding in all generations has mostly used a scFv, linked via a hinge domain (mostly derived from IgG C_H1_C_H2_ or C_H2_C_H3_ regions) to a transmembrane region (mostly from CD3ξ) and a cytoplasmic region for TCR signaling from CD3ξ. The second generation added an intracellular costimulatory domain, and the third generation added two costimulatory domains. The costimulatory domains were usually CD28, CD137 (4-1BB/TNFRSF9), or CD134 (OX40). The fourth generation (TRUCKs) are engineered to release an inducible payload, usually IL-12, and may also contain a controllable on-off switch, or suicide gene. i = ITAM (immune receptor tyrosine-based activation motif).

CARs are often delivered to T-cells with lentiviral vectors derived mainly from the HIV-1 genome. These vectors stably integrate their RNA genome into host cell genomes of dividing or non-proliferating immune cells in the form of a cDNA copy that is reverse transcribed into DNA in the target cell [331,332]. Lentiviruses carry a lower risk of insertional mutagenesis compared with other retroviruses that can only infect dividing cells, since they integrate into the host genome at sites away from gene promoters [333,334]. Other methods of nonviral gene transfer such as sleeping beauty transposon and clustered regularly interspaced short palindromic repeats (CRISPR) are being investigated [334,335]. CAR-T design has undergone several generational changes since they were first developed in 1989 [336]. See Figure 3 for the different generations of CAR designs. Their architecture consists of several parts: (1) a leader peptide/signal sequence to direct the protein to the cell surface; (2) the ectodomain/extracellular region with a segment for antigen recognition and binding, most commonly consisting of an scFv; (3) a spacer/hinge; (4) a transmembrane domain, usually derived from CD3ζ; (5) an endodomain/intracellular region with a signaling domain. The signaling domain relies on the phosphorylation of ITAMs, most commonly from CD3ζ, to mediate T-cell activation (signal 1). The spacer/hinge and transmembrane regions were originally viewed as inert structural links between the extracellular and intracellular regions, but variations in each have been found to affect their function [329]. The second generation of CARs incorporate additional intracellular co-stimulatory signaling domains (signal 2), including CD28 or CD137 (4-1BB/TNFRSF9), to improve persistence of the T-cells in vivo by reduction of anergy and activation-induced cell death [77]. This generation includes Axicabtagene ciloleucel (Yescarta) and Tisagenlecleucel (Kymriah) which target CD19, the first CAR T-cell cancer immunotherapeutics to gain FDA approval, in 2017 [337]. The third generation CARs have two or more costimulatory domains (e.g., CD28, CD137, or OX40/CD 134 for enhanced T-cell proliferation and cytotoxicity) [77]. The fourth generation, also known as T-cells redirected for universal cytokine-mediated killing (TRUCKs) [338], include additional CAR T-cell improvements to enhance T-cell function and enrichment and minimise senescence. Enhancements may include chemokine receptors and/or cytokine transgenes with inducible expression and release of their payload (e.g., IL-12 a cytokine with anti-tumour activity and important for the regulation of adaptive T-cell responses), a controllable on-off switch, or suicide gene [261,339]. Bispecific CARs can simultaneously target 2 antigens/epitopes [336,339,340].

The starting cell population used for many CAR T-cell therapies consists of CD4+ and CD8+ T-cells at the ratio present in the peripheral blood of the patient [341]. However, as seen with adoptive cell immunotherapies, the ratio and type of T-cell subsets selected for use is a factor critical for their success and contributes to the differences in efficacy and toxicity profiles of the different CAR-T-cells [342]. T-cells exhibit clear differences in effector function, proliferation, and metabolic requirements. Increasing levels of stimulation induce differentiation and transition from naïve T-cells into stem-cell memory T, then into central-memory T, effector-memory cells, and finally into terminally differentiated effector-T-cells with decreased production of IL-2 and reduced proliferation. Both CD4+ and CD8+ CAR T-cells derived from naïve or central-memory T-cells exhibit a more potent antitumor activity in vivo compared with effector-memory T-cells [334].

The first two CAR T-cell therapies for commercial use employed autologous T-cells for patients with B-cell malignancies. In both cases, the antigenic target for these CAR-T therapies was CD19, a transmembrane protein confined to the B cell lineage and present on most B-lineage lymphomas and leukaemias [330]. Despite B-cell depletion (aplasia), significant on-target off-tumour toxicity occurred, with approximately 80% of patients experiencing cytokine-release syndrome (CRS). CRS is associated with massive release of tumour cell components and pro-inflammatory cytokines into the blood stream, monocyte/macrophage activation and risk of multiple organ failure, intensive care unit admission and additional therapeutic interventions [324,343]. Data suggest that CAR-T-associated CRS depends on T-cell engagement with target antigen [344]. For solid tumours, the risk of CAR-T- associated CRS is lower, although as with haematological cancers, the magnitude of CRS correlates with the tumour burden. Instead, on-target/off-tumour effects seen with CAR-T for solid cancers are due to targeted antigens being not unique to the cancer. Attempts to limit unwanted CAR-T toxicity have driven further CAR-T therapy changes with the introduction of simultaneous targeting of 2 different antigens. This has been achieved with (1) tandem CARs, with two different activating CARs recognising two different tumour antigens on the same cell, one with a CD247 signaling domain and the other with a CD28 co-stimulatory domain, or (2) inhibitory CAR (iCAR), with dual antigen recognition, but for a single tumour antigen with a signaling and co-stimulatory domain, plus a normal antigen with an inhibitory PD-1 or CTLA-4 signaling domain. Both approaches aim to enhance selective tumour cell eradication while protecting normal cells expressing only one of the antigens [151].

Solid tumours present physical barriers to CAR-T cells reaching their target. For example, intravenously injected T-cells require trafficking and homing to the correct location for efficacy. They must then undergo extravasation and movement through the ECM, stroma, and TME with their additional obstacles before reaching the malignant cells [117]. More direct administration near to the site of the tumour, e.g., intraperitoneal (i.p.) administration for ovarian cancer [345], may therefore be required. In support of this, a xenograft mouse model of ovarian cancer with CAR-T cells targeting tumour-associated glycoprotein (TAG72), found that i.p. delivery eliminated antigen-positive disease and extended the overall survival of mice, while intravenous CAR-T cell delivery was ineffective in controlling disease [346]. Further improvements to CAR-T design for solid tumour immunotherapy will be required to overcome immunosuppressive influences from the TME that result in suboptimal CAR-T cell function. Such influences include release of inhibitory cytokines and depletion of the essential amino acid tryptophan by TAMs and MDSCs, while design improvements may include co-expression of chemokine receptors, or soluble cytokines [77].

The reliance of original versions of CAR-T therapies on patient-derived autologous T-cells recognising tumour antigens has limited their use for various reasons. These include a lack of T-cells in patients with chemotherapy-induced lymphopenia, limited targeting of only one or two epitopes, the need for a skilled team with access to a good manufacturing process-compliant facility with substantial investment in processing infrastructure, delays in patient treatment and variable clinical outcomes [347]. Allogeneic T-cells from healthy donors on the other hand should provide more consistent results in terms of T-cell potency, but this needs to be balanced with the prevention of dangerous off-tumour anti-host activity through graft versus host disease (GVHD) due to recognition of alloantigens in the recipient. In addition, prevention of rejection of the foreign T-cells due to expression of different HLA antigens on their surface needs to be overcome [348]. Different gene-editing proteins have been used to edit the constant regions of the TCR α or β chains, or to disrupt the HLA-A locus of the MHC gene complex of the donor T-cells to interfere with TCR function or MHC class I expression to generate “universal” T-cells and eliminate off-tumour effects. These include zinc finger nucleases (ZFNs), transcription activator-like effector nucleases (TALENs), meganucleases (MNs), and CRISPR with Cas9 nuclease [340,349].

Other options for generating universal, off-the-shelf CAR-T cells, incorporate new CAR-T designs that use a “third party” intermediate with a lock and key mechanism that splits the antigen-targeting domain from the T-cell signaling domain [340]. This system allows swapping of antigen targets without affecting the T-cells used. One such lock and key design relies on biotin-avidin binding; with biotin-labelled antigen-specific molecules able to bind avidin linked extracellularly to a transmembrane domain and intracellular signaling domains [340]. Another lock and key design uses separate leucine zippers that bind each other, with one attached to an antigen binding scFv and the other linked to the T-cell CAR. The structures of the leucine zippers that bind each other can be adjusted so they bind each other with varying affinities, thus allowing some control of the potency of T-cell signaling and activation [340].

#### 6.8.3. Alternative Adoptive Cell Therapies: DCs, Gamma-Delta (γδ) T-Cells, Fcγ-CR-T-Cells

An alternative to TILs or T-cells for adoptive cell transfer is provided by dendritic cells and has been investigated in preclinical and clinical studies. The presence of APCs is generally low in the TME. Increasing the number of effective APCs at the tumour site would increase the potential for augmenting the immune response against cancer cells by cross-presentation of TAAs to CD8+ cells [261], and to shift the balance from immunosuppression to immune surveillance and elimination [4]. Autologous DCs generated by various protocols have been trialled, but the most popular approach uses peripheral blood monocytes treated with GM-CSF and IL-4, or TNFα in vitro. Vaccination with these DCs is combined with loading or pulsing with either tumour cell lysates or more specific antigen or tumour-derived peptides [350]. From 2000 to 2015, there were 13 clinical trials with 148 patients receiving DC immunotherapy for ovarian cancer targeting antigens that included MUC1, MUC16, HER2, and FOLR1, with variable and inconsistently reported results [4]. A more recent study included five recurrent ovarian cancer patients with DCs loaded with hypochlorous acid-oxidized whole tumour lysate (to induce primary necrosis and enhance the immunogenicity of lysed tumour cells), administered into the regional lymph node (intranodally). Two patients had a durable progression-free survival for two years or more [269]. Cancer vaccines, including DC vaccines, however, have been criticised for their low therapeutic efficacy, so it is thought that combining them with other immunomodulatory agents such as immune checkpoint inhibitors, IDO1 inhibitors, Ontak, and other intervention strategies may improve patient outcomes [269]. Allogeneic DC vaccines [261] and DC-derived exosomes [351] have been trialled for some cancers, though to date not ovarian cancer.

T-cells whose TCRs are composed of heterodimers of glycoprotein γδ chains instead of αβ chains (gamma-delta T-cells) are at the border of the innate and adaptive immune system. Like conventional TCRs, they rearrange their TCR gene segments, but in a restricted way for pattern recognition and develop a memory phenotype. They normally make up only about 2% of T-cells and are found mostly in the gut mucosa as intraepithelial lymphocytes [352]. However, they are also found in other epithelial and mucosal barrier sites. In contrast to cells with TCR αβ, those with TCR γδ bind directly to an antigen’s superstructure and are independent of MHC/peptide complexes [353]. They can also produce inflammatory cytokines, directly lyse infected or malignant cells, and establish a memory response to attack pathogens upon re-exposure [353]. They have been found in the peritoneum [354] and ovarian cancer TILs [352]. A recent study looking at expression profiles of thousands of tumour samples from various cancer types has shown that overall survival correlates with high levels of intra-tumoural γδ T-cells [355]. These properties make them candidates for the basis of universal allogeneic T-cell therapies [353]. However, it has also been shown that specific subsets of γδ T-cells can directly promote cancer progression through secretion of immunosuppressive IL-17, while others can inhibit DC maturation or induce DC senescence [352]. A clinical grade protocol to isolate and expand large numbers of polyclonal γδ T-cells capable of gene-modification to enhance anti-tumour activity has been developed recently [356].

Fc gamma chimeric receptors (Fcγ-CRs) are similar to CAR-T based immunotherapies, though the CAR scFv recognising a targeted TAA is replaced by the extracellular portion of the FcγRIIIA (CD16). Together with therapeutic mAbs, they enhance anticancer activity by an antibody-mediated cellular cytotoxicity mechanism triggering downstream activating pathways resulting in perforin/granzyme-dependent tumour cell lysis [357].

### 6.9. Targeting the Glycocalyx

Targeting the glycocalyx or extracellular carbohydrate component of tumour proteins to disrupt their ligand binding interaction with Siglecs on immune cells is a strategy that can be exploited for cancer immunotherapy [56,58,195]. Cell surface sialosides play a major role in immune modulation that is utilised by tumours to evade destruction by the innate and adaptive immune system [58]. Sialidase (neuraminidase) treatment of a variety of cancer cells removes Siglec-7 and Siglec-9 ligands on cancer cells in vitro and promotes cancer cell killing by NK cells [56]. Sialidase-treated cancer cells injected i.p. into mice with human NK cells expressing high levels of Siglec-7 were cleared faster than untreated cancer cells [358]. More precise glycocalyx editing by simultaneous antigen targeting has been developed and tested in vitro. A recombinant sialidase enzyme derived from *Vibrio cholera* conjugated to trastuzumab (targeting HER2) was found to selectively desialylate HER2-positive breast cancer cells, thereby reducing ligands for binding by inhibitory siglecs-7 and -9 on NK cells. At the same time, potentiated NK-cell mediated killing was enhanced by increased binding of the NK cell activating receptor NKG2D (KLRK1 gene) to its ligands present on the cancer cells [58].

### 6.10. Overcoming Immune Suppression of the TME

Alternative strategies for immunotherapies to overcome immune suppression of the TME may include reprogramming the altered metabolism of TILs and targeting Tregs directly at the tumour site. Metabolic strategies include enforced expression of PGC1α to reinvigorate dysfunctional T-cells for cancer treatment [156] and targeting IDO1 production by cancer or myeloid cells. Epacadostat, an orally available small molecule IDO1 inhibitor, is included in current trials for ovarian cancer to relieve Treg cell-mediated immune tolerance [87]. One trial investigated epacadostat with MSLN in patients with platinum-resistant ovarian, fallopian tube, or peritoneal cancer, [247]) (NCT02575807), but the study was terminated due to low enrolment and lack of clinical activity [246]). One current trial is investigating epacadostat together with a fusion protein targeting NY-ESO-1 in dendritic cells in patients in with epithelial ovarian cancer (NCT02166905). Another trial is testing epacadostat with conventional therapeutic surgery with patients with newly diagnosed stage III-IV epithelial ovarian cancer (NCT02042430). Tregs themselves may be targeted with antibodies against CD25 or CTLA-4 and/or OX40 which are all constitutively expressed in these cells [54]. Alternatively, the secreted products of Tregs may be targeted, e.g., TGFβ, IL-10, IL-35 [51], or their receptors, e.g., chemokine receptor 8 (CCR8) [359].

TAMs provide alternative opportunities for immunotherapy. Preclinical studies have shown that the primary tumour growth and the number of metastatic sites can be significantly reduced by decreasing the population of macrophages in tumour tissue, e.g., by blocking recruitment of monocytes or eliminating TAMs already present, reprogramming TAMs into proinflammatory M1 macrophages or neutralizing the protumoral products of TAMs [360]. A potential future immunotherapeutic strategy could use TAMs for anticancer drug delivery into the tumour environment. Nanoparticles have been introduced into clinical trials to enhance local drug accumulation and reduce systemic toxicity [361]. Mouse studies that included i.p. injected human ovarian cancer cells have shown that polymeric nanoparticles incorporating platinum accumulate in TAMs, then deliver their payload to neighbouring tumour cells and inflict DNA damage [361]. In addition, human macrophages have been genetically engineered with CARs to boost antigen-specific phagocytosis and tumour reduction in pre-clinical models [321].

## 7. Additional TAAs as Immunotherapeutic Targets for Ovarian Cancer

In addition to the general immune and TME targets for therapy applicable to many cancer types as discussed in previous sections, a large number of different TAAs are expressed in ovarian cancer that have been targeted in clinical trials and shown in Table 3. Apart from NY-ESO-1 and other CTA antigens, these include MUC16, mesothelin, and folate receptors [336], as well as antigens not widely expressed outside of the reproductive system (e.g., follicle-stimulating hormone receptor, FSHR) [362].

### 7.1. MUC16 and MUC1

MUC16 is the most commonly presented peptide antigen in ovarian cancer. [30]. It is the largest protein of the mucin family, 22,152 amino acids [363], and is the second longest human protein. Transmembrane mucins extend between 200 and 500 nm above the plasma membrane, compared to most membrane glycoproteins, which usually protrude < 30 nm [364]. MUC16 has a high predicted molecular weight (2353 kDa) [192], with a very long, hyperglycosyalted extracellular domain with a multiple tandem repeat and a relatively short cytolplasmic tail linked to the actin cytoskeleton [365]. It has 249 potential N-glycosylation sites and more than 3,700 O-glycosylation sites [363], and its carbohydrate content accounts for up to 77% of its weight [364]. It is normally produced by the epithelium of the fallopian tubes, endometrium, endocervix, cornea, conjunctiva, trachea, and bronchi [192,366], and has been detected in mesothelial cells of the pleura, pericardium and peritoneum. It is overexpressed in most epithelial ovarian cancers and subsets of other cancers, and a soluble form shed into the plasma is produced by cleavage from the membrane-bound form of MUC16 [365]. The STn glycoform has recently been found to be a better indicator of tumour burden and relapse, and prognostic marker for HGSOC compared to conventional serum CA125 [367]. Ligands for MUC16 are mesothelin (MSLN) as well as Siglec-9 on the surface of NK cells [86]. Binding between the membrane bound form of MUC16 and Siglec-9 prevents the formation of an immunological synapse between tumour cells and NK cells, thereby preventing the cytotoxic activity of NK cells [86,194,368]. The shed form of MUC16 also behaves as a decoy for Siglec-9 interactions with NK cells, B cells and monocytes [173]). Two mAbs against MUC16, oregovomab and abagovomab, have been trialled separately in phase III clinical trials in patients with ovarian cancer, but neither showed significantly improved clinical outcomes compared to controls [365]. Their failure is thought to be due to soluble MUC16 acting as a sink for mAb binding and failure of the mAb to reach the cancer cells. In addition, therapeutic efficacy could be reduced by shedding of MUC16 after mAb binding to membrane-bound antigen. An antibody drug conjugate (DMUC5754A) uses an alternative MUC16 antibody directed against the repeat region conjugated to a drug (monomethyl auristatin E; MMAE) that disrupts microtubules, and was found to have an acceptable safety profile and some evidence of anti-tumour activity in a Phase I clinical trial for ovarian cancer patients with platinum resistance [365]. An alternative antibody drug conjugate also targets MUC16 with MMAE, but uses an alternative conjugation technology to achieve a consistent drug-to-antibody ratio (DAR) of 2:1 instead of the usual heterogeneous mix of 0–8:1. This was tested in a Phase I clinical trial in patients with platinum-resistant ovarian cancer, and found to have an acceptable safety profile with evidence of anti-tumour activity, and is expected to undergo further evaluation for ovarian cancer [83].

Most MUC16 targets have been directed against epitopes in the extracellular cleaved portion of the molecule. The remaining membrane bound extracellular portion of the molecule provides an alternative immunotherapeutic target that is not subject to the negative sink/decoy effect provided by the bulk of the MUC16 molecule. An armoured MUC16 CAR (4H11-28z/IL-12) with a safety elimination gene has been developed for autologous T-cells that targets an extracellular portion of the protein that is retained after cleavage and not glycosylated (MUC16ecto), secretes IL-12 and contains a truncated portion of EGFR. The EGRFt component is included so that any on-target, off-tumour effects or other toxicities may be reduced by administration of cetuximab (an EGFRt mAb) [345]. A phase I clinical trial is currently underway (NCT02498912), and due to be completed in 2020.

Tumour associated MUC1 (TA-MUC1), is expressed in >90% of epithelial ovarian cancers and associated with cancer progression properties, such as tumour stage, grade, residual disease status and presence of ascites [369]. It has been targeted with the mAb gatipotuzumab in patients with advanced ovarian cancer, with variable results and some clinical benefit seen in almost half of patients [191].

Targeting the glycocalyx and aberrant glycoforms present at the cell surface to overcome barrier effects and disrupt negative cancer-immune cell signaling in the ovarian TME have potential to be important in future immunotherapy. Less precise targeting of soluble or membrane bound MUC16 and other elements of the ovarian cancer TME, may be provided by alternatives to i.p. sialidase enzyme-antibody conjugate treatment. The high density of O-glycosylation on mucin domains makes them resistant to digestion to common proteases such as trypsin. A protease derived from *Escherichia coli*, however, was found to have a distinct peptide- and glycan-based cleavage motif that enables high selectivity for mucins, and has been used in vitro to digest cancer-associated mucins. These include those from cultured cells (MUC1 from breast cancer and chronic myelogenous leukaemia cell lines, and MUC16 from a cervical cell line), and ascites from ovarian cancer patients [181]. An alternative enzymatic treatment may be provided by bromelain, that contains several endopeptidases and glucosidases (and many other factors). It also has anti-inflammatory and antioxidant effects, is effective for removing necrotic tissue and treating wounds and importantly is safe to use in humans. It has FDA approval for clinical use [370,371,372]. In rats, i.p. administration of bromelain prevents intra-abdominal adhesions [371], and when combined with N-acetyl cysteine (NAC, a mucolytic agent also used clinically) to treat rats with pseudomyxoma peritonei, a malignancy in which excessive mucin secreted by tumour cells accumulates in the peritoneal cavity, mucin disintegration was observed without any toxicity [370]. A clinical trial with bromelain and NAC (NCT03976973) was registered recently for patients with mucinous peritoneal tumours. Antitumoural effects of bromelain have been found with increased survival in mice with various cancer cell lines, including an ascitic mouse cell line (Ehrlich ascitic tumour) [373], and in vitro with a human cancer cell line (A2780) [374]. (Like SKOV3, although A2780 is used as a model of ovarian cancer, it is unlikely to represent HGSOC [132]). Silica nanoparticles modified by conjugation with bromelain with the aim of overcoming the mucous barrier were found to have increased proteolytic activity towards the ECM of in vitro tumours [375].

### 7.2. Tumour-Associated Glycoprotein 72 (TAG72, Sialyl-Tn)

In combination with hypersialylation found in cancers [56], *O*-Linked Tn, Sialyl-Tn and T antigens are produced by incomplete glycosylation of mucins and are characteristic for most carcinomas [183]. Sialyl-Tn has been identified as tumour-associated glycoprotein 72 (TAG72) and is present in high levels in multiple histological sub-types of ovarian cancer [346]. The presence of TAG72 has been confirmed on MUC1 and CD44 [376] (an ovarian cancer stem cell marker and receptor for the ECM molecule hylauronan [377]) as well as CD133 (a marker present in cancer stem-cells including ovarian cancer) [378] and MUC16 proteins [379] in ovarian cancer patients. CAR-T cells engineered with a CD137 co-stimulatory signaling domain targetting TAG72 have shown cytokine production and cytoxicity against TAG72+ ovarian cancer cell lines and ascites [346]. In addition, i.p. delivery of these cells significantly reduced tumour growth and improved overall survival in mice with peritoneal ovarian cancer xenografts [346].

### 7.3. Mesothelin (MSLN)

The interaction between MUC16 and MSLN contributes to peritoneal adhesion and spheroid formation, and provides a targeting strategy under development to reduce peritoneal metastasis and facilitate the use of other therapies [365]. MSLN is a transmembrane glycoprotein relatively specific to mesothelial cells that line body cavities including the peritoneum, but is also highly expressed in many tumour cells including mesothelioma, ovarian and pancreatic cancer. A soluble form is also present in the circulation of these patients [365]. It is the most common peptide antigen presented by MHC-II in ovarian cancer [30]. A number of agents including CAR T-cells targeting MSLN have been developed, and are currently in phase I and II clinical trials for ovarian cancer [380]. Amatuximab (MORAb-009) is a mAb that mediates inhibition of MSLN-dependent cell adhesion and antibody-dependent cellular cytotoxicity, and can block the binding of MSLN to MUC16 [313]. An antibody–drug conjugate anetumab ravtansine/BAY94-9343 targets MSLN with a mAb conjugated to an anti-mitotic tubulin inhibitor DM4 [313]. Pre-clinical ovarian cancer models have been favourable but only a small number of ovarian cancer patients have been included in trials [313]. Another antibody–drug conjugate in a clinical trial currently underway for patients with advanced solid tumors including ovarian cancer, uses a mAb targeting MSLN conjugated to a cytotoxic drug BMS-986148 with and without Nivolumab (NCT02341625) [381]. A variation of this antibody–drug approach to MSLN-directed immunotherapy uses recombinant fusion protein immunotoxins. SS1P is an immunotoxin consisting of a MSLN-targeting scFv disulphide-linked to a *Pseudomonas* toxin, which is internalised by the cell through endocytosis and has been tested as a single agent as well as in combination with chemotherapy [247,313]. However, on-target off-tumour toxicity limits the dosage and efficacy is reduced by formation of antibodies against the drug. This prompted development of a similar immunotoxin with a less immunogenic *Pseudomonas* toxin (LMB-100), which has been trialled on patients with mesothelioma and pancreatic cancers [247]. Another vaccine using an engineered bacterial agent, CRS-207 has been designed to secrete MSLN that is delivered to APCs. It is live-attenuated *Listeria monocytogenes* bacteria with deletion of two virulence factors that reduce its toxicity, while maintaining potent innate and adaptive immunity stimulation. CRS-207 has been trialled in small numbers of platinum-resistant ovarian cancer patients (phase 1 and 2) with and without pembrolizumab, and epacadostat (NCT02575807) [247]. The trial, however, was terminated due to low enrolment and lack of clinical activity [246]. In separate clinical trials with CRS-207 for other conditions, two patients out of more than 350 developed listeriosis, leading to a partial hold by the FDA in late 2016 of all trials with this vaccine [382]. Despite some success in one clinical trial in mesothelioma in combination with chemotherapy with objective tumour responses in the majority of patients [383], the company responsible for its development stopped its clinical program with CRS-207 [384]. There are also a number of clinical trials currently underway which include ovarian cancer patients with anti-MSLN CAR-T cells [365].

### 7.4. Folate Receptor Alpha (FRα, FOLR1 Gene)

Folate receptor alpha (FRα, gene FOLR1) binds folic acid with high affinity and transports folate (vitamin B9) by receptor-mediated endocytosis. It has restricted expression in normal cells but is highly-expressed in various non-mucinous tumours of epithelial origin, including ovarian cancer, where increasing levels of tissue FRα are associated with tumour progression from early to advanced stages making it an attractive therapeutic target [385]. High FRα expression in serous ovarian cancer is also correlated with poorer response to chemotherapy, shorter disease-free survival, and reduced overall survival compared to low FRα expressing tumours [203]. Although peptide fragments of this protein have been found to be presented on MHC class I molecules from approximately 24% epithelial ovarian cancers, such fragments were also found in approximately 17% benign ovarian/fallopian tube samples in the same study, though absent in other benign tissues (liver, kidney, colorectal) and normal PBMCs [30]. Farletuzumab (MORAb-003) is a FRα-targeting mAb tested in combination with standard platinum-based chemotherapy in patients with relapsed platinum-sensitive ovarian cancer patients in a Phase 3 clinical trial. Overall, the treatment failed to improve progression-free survival compared to the control group. However, patients with a low serum MUC16 showed improved progression-free and overall survival [386]. These results were explained by direct binding of MUC16 to farletuzumab that prevents Fc-γ receptor engagement on effector cells such as NK cells, thereby suppressing the beneficial effect of ADCC [192]. Mirvetuxemab soravtansine is a well-tolerated [315] antibody–drug conjugate consisting of a mAb linked to an anti-mitotic agent, maytansinoid DM4, to target ovarian cancer cells expressing FRα. Preliminary results from a trial with recurrent, platinum-resistant ovarian cancer (17 objective tumour responses from 37 patients), have encouraged further clinical trials which are currently ongoing [300].

### 7.5. Follicle-Stimulating Hormone Receptor (FSHR)

FSHR has been tested in preclinical models as an immunotherapeutic target since it is expressed in gynaecologic malignancies, including serous and other ovarian cancers, but not in non-ovarian healthy tissues [387]. A variant of CAR has been used in which T-cells are engineered to express the ligand for a hormone receptor, i.e., FSH, instead of an antibody fragment to find the target, FSHR. When tested in mice these modified T-cells were able to induce rejection of human ovarian cancer cells in immunocompetent animals, without any adverse events [387]. A more recent preclinical study with immunocompetent mice implanted with syngeneic epithelial ovarian tumour cells, used an engineered DNA vaccine against FSHR. Untreated mice developed aggressive peritoneal carcinomatosis, however the vaccine increased the presence of TILs and promoted generation of FSHR-specific T cells, and survival of the mice [388].

## 8. Potential Advances

### 8.1. Optimising Existing Therapies, Adaptive Therapy

Problems with resistance to existing immunotherapies due to genomic instability and the requirement for effective treatment combinations are driving development of alternative targets and strategies for cancer immunotherapy. The optimal timing, dosing, and sequencing of immunotherapies with chemo- and other therapies remains to be determined [389], with the potential aim of cancer control with increased long-term survival rather than cure [390]. This could be achieved with adaptive therapy that allows for survival of a significant population of (chemo)sensitive cells, which in turn suppresses proliferation of less fit but (chemo)resistant subpopulations, instead of more standard treatment with or near to the maximum tolerated dose of chemotherapy. This was demonstrated previously with severe combined immuodeficient mice implanted with cells from the HGSOC OVCAR3 cell line, and subsequently treated with decreasing carboplatin doses based on the change in tumour size. These mice based survived for the duration of the experiment with substantially decreased tumour burden compared to those treated with the standard carboplatin treatment based on the maximum tolerated dose [391].

### 8.2. Enhancing Immunotherapy with Epigenetic Modulating Therapy

Epigenetic dysregulation is a key mechanism contributing to cancer development, immune evasion, and progression [392]. The expression of a number of CTAs including NY-ESO-1, is regulated epigenetically, with upregulation of expression occurring after exposure to demethylating and deacetylating agents [203]. Epigenetic modulation of serous ovarian tumours with TAAs whose expression is epigenetically regulated and heterogeneous, may be an option to improve the outcomes of such TAA-targeting immunotherapies [203]. Decitabine (DAC), a deoxycytidine analog and DNA hypomethylating drug that inhibits DNA methyltrasferase (DNMT), is approved for the treatment of myelodysplastic syndrome and synergises with platinum-based chemotherapy drugs including carboplatin to activate transcription of epigenetically silenced (hypermethylated) genes [393]. DAC treatment upregulated NY-ESO-1 and MHC class I expression in ovarian cancer cell lines in vitro with the expectation of increased recognition by antigen-reactive CD8+ T-cells [203]. DAC similarly increased expression of protein expressed in prostate, ovary, testis, and placenta (POTE) genes in ovarian cancer cell lines [139]. POTE genes are a subfamily of 14 genes from the CTA family, localised to autosomal pericentromeres, with increased expression and DNA hypomethylation of POTEs C, E, and F in HGSOC [139], which may lend themselves to future epigenetic modulation in conjunction with immunotherapy. However, it has been reported that DNA methylation patterns vary across the histologic subtypes of ovarian cancer, and that HGSOC has relatively fewer DNA hypermethylation changes than endometrioid and clear cell ovarian cancers [394].

Besides the upregulation of expression of TAAs, DNMT inhibitors and histone deacetylase (HDAC) inhibitors are able to upregulate expression of other immune signaling components of cancer cells. These include components of antigen processing and presentation machinery, surface expression of co-stimulatory molecules, stress-induced ligands, death-inducing receptors, and immune checkpoint ligands [392].

Epigenetic mechanisms also regulate normal, physiological differentiation of immune cell lineages, including CD4+ T-cells naïve to effector CD8+ T-cells and myeloid cells [136] as well as NK cells [395,396]. In addition, cancer-associated T-cell exhaustion is regulated by epigenetic processes including DNA methylation, histone modifications and alterations to chromatin conformation that may be amenable to therapeutic interventions such as DNMT inhibitors [136].

### 8.3. Exploiting the Innate Immune System

Therapies that increase NK cell frequency, function, and/or migration into tumours have the potential to complement T-cell based immunotherapies [114]. Likewise, strategies similar to those used for T-cell immunotherapies are expected to provide some success [114,397].

#### 8.3.1. Adoptive and Engineered NK Cell Therapy

NK cells, usually defined as CD56+, but also characterised by the CD16 surface antigen (low affinity, activating FcγRIII), are innate immune effector cells that do not express TCRs or CD3. They have the ability to exert rapid cytotoxicity against virus-infected and cancer cells with generation of cell debris for capture by APCs without prior antigen sensitisation due to their ability to target and kill cells independent of MHC [113]. As such, they should be well suited for adoptive immunotherapy [398], and offer opportunities to produce an off-the-shelf product available for clinical use [113]. However, attempts with adoptive NK cell immunotherapy against ovarian cancer have proven unsuccessful, with the main limitations including failure to expand and diminished effector function. In order to address this, in vitro activation of human NK cells with IL-12, IL-15, and IL-18 has been found to result in enhanced IFNγ production with effective killing of cancer cells in vitro as well as in an in vivo mouse model of ovarian cancer [398].

Like T-cells, expression of CARs in exogenous NK cells has enabled NK recognition of tumour- associated antigens with increased survival, proliferation and cytotoxicity [399]. Advantages of NK cells include: (1) the lack of GVHD due to their reaction to lowly expressed or absent MHC rather than mis-matched MHC; (2) their relatively limited life-span, permitting effective antitumor activity while reducing the probability of long-term adverse events, due to on-target/off-tumor toxicity to normal tissues); (3) CAR-NK cells retain their intrinsic capacity to recognise and target tumor cells through their native receptors [400]. An alternative MSLN-targeting CAR therapy has been tested successfully in vitro and in vivo in a mouse model of ovarian cancer. It relies on induced pluripotent stem cells to produce a renewable source of NK cells engineered to express CAR constructs, and provides a potential method for producing “off-the-shelf” allogeneic cells for immunotherapy [401].

The relatively recent realisation that NK cells and other innate immune cells, including macrophages, and dendritic cells, can acquire memory properties that confer broad, non-specific immunological protection against future immunological threats [402,403] will lead to implementation of new immunotherapeutic approaches for cancer [403]. This “trained immunity” [404], can be brought about by in vitro conditioning, e.g., with BCG, and cytomegalovirus (CMV) [403], with altered intracellular metabolism‘, and epigenetic changes/chromatin modifications that affect gene transcription [402] after PAMP/DAMP recognition by PPRs [403]. It is expected that care will be required to avoid concurrent unwanted pathological inflammation [403].

Similar to BiTes, bi-, tri-, and tetra-specific killer engagers (BiKEs, TriKEs, TetraKEs) have been engineered to form an antigen-specific immunological synapse between tumour cells and NK cells via an anti-CD16 component instead of CD3 with the aim to effect NK cell-mediated killing of their tumour targets [397]. Later versions include IL-15 in their design to enhance NK function, and targets have included HER2, EpCAM, and CD133 [397].

#### 8.3.2. NK Cell ICB

Similar to T-cell checkpoint blockade, blocking negative regulators of NK cell function should be a successful strategy for cancer immunotherapy [114,397,405].

Blocking cell surface receptors bearing ITIMs that normally deliver an inhibitory signal to immune effector cells including NK cells is expected to be a useful strategy for immunotherapy on its own and to enhance more conventional T-cell ICB [405]. Killer immunoglobulin-like receptors (KIRs) are commonly expressed NK cell receptors. Some are activating but others are inhibitory due to the presence of ITIMs, so are the focus as promising targets for ICB [397]. The CD94-NKG2A heterodimer is another inhibitory receptor expressed on many circulating NK cells, as well as CD8+ T-cells [397]. An antibody to NKG2A (monalizumab) has been used in vitro and in clinical trials to enhance the activity of NK cells and CD8+ T-cells, and it is expected that combining the blockade of inhibitory signals with the delivery of activating signals should improve the efficacy of immunotherapies [405].

TGFβ signaling is a suppressor of NK cell function, with inhibition of NK metabolism, proliferation, cytotoxicity, cytokine production, and antimetastatic functions in various mouse models of cancer [114]. The expected protective effect of tumour infiltration by NK cells is lost during TGFβ-mediated epithelial–mesenchymal transition (EMT), and inhibitors of this pathway promote control of epithelial-derived tumours in pre-clinical models. Signaling through activin-A (a dimer of inhibin-β and a member of the TGFβ superfamily) mediates TGFβ-like immunosuppressive effects and may provide a basis for future immunotherapy enhancement [114].

#### 8.3.3. Phagocytosis

Another alternative to ICB of T-cells or NK cells, is myeloid-specific checkpoint inhibition, e.g., by targeting the CD47/signal regulatory protein α (SIRPα) axis which regulates macrophage activation and phagocytosis. CD47 (Integrin-associated protein, IAP) is a transmembrane protein widely expressed in normal tissues and seen as a marker of “self”. It transduces inhibitory signals through SIRPα on macrophages and other myeloid cells. Its overexpression on cancer cells is regarded as a “don’t eat me” signal for phagocytes, and in ovarian cancer its mRNA expression is associated with adverse outcome. Blocking this pathway enhances phagocytosis by macrophages, cytokine secretion and antigen uptake and presentation. It may also synergise with T-cell checkpoint inhibitors [406]. A clinical trial with a mAb against CD47 (Hu5F9-G4) in combination with anti-PD-L1 (Avelumab) is currently underway for ovarian cancer patients who progressed within 6 months of prior platinum-based chemotherapy (NCT03558139) [224,407]. Another (phase I trial; NCT03957096) that includes epithelial ovarian cancer patients, uses a CD47 antibody–drug conjugate (SGN-CD47M) [225].

An additional “don’t eat me” signal, and perhaps the most dominant innate immune checkpoint for ovarian cancer has recently been identified as CD24 [408]. CD24 is a small sialoglycoprotein expressed on malignant cells where it is thought to be a cancer stem cell marker [409]. It is also present in haematopoietic, epithelial, and other cells [410]. It is a promising target for blockade for ovarian (and breast) cancer immunotherapy [408]. Its upregulation of expression on ovarian cancer cells compared to normal cells is greater than that for both CD47 and PD-L1, and is greatest for ovarian cancer when compared to numerous other cancers [408]. Low CD24 expression is associated with increased relapse-free survival for ovarian cancer patients. The cellular target for CD24 is Siglec-10 present on TAMs in ovarian cancer, but expressed at low levels on peripheral blood mononuclear cells and peritoneal macrophages from patients without cancer. In vitro experiments have shown that phagocytosis by donor-derived macrophages is increased significantly against ovarian cancer patient cells treated with a CD24 mAb compared to CD47 mAb treatment and control [408].

A dual-CAR approach targeting CD24 and MSLN with engineered NK cells has been tested in vitro against ovarian cancer cell lines and patient-derived primary tumour samples with promising results [411].

### 8.4. Miscellaneous Alternatives: Nanobodies, Engineered Bacteria, Systemic CD8+ T-Cell-Mediated Immunity

Potential problems with CD47 blockade include anaemia and thrombocytopaenia due to the high expression of CD47 on erythrocytes and platelets. An attempt to improve the therapeutic profile and limit potential side effects of anti-CD47 therapy have included two innovations: the use of a nanobody with higher binding affinity than a mAb against CD47, and bacteria engineered to colonise tumours and undergo synchronised lysis [412].

In contrast to scFvs, nanobodies (Nbs, also known as VHH) are approximately half the size (15 kDa) and are derived from the variable region of the heavy chain of camelid antibodies. Camelid antibodies lack light chains and are themselves approximately half the size (75 kDa) of conventional mAbs [413]. Compared to conventional mAbs they have reduced immunogenicity, improved thermal and chemical stability as well as higher solubility [413,414]. Their small size allows great flexibility in terms of engineering options, with selection of high affinity targeting [414], and enable faster and greater tumour penetration through leaky tumour vasculature compared to conventional antibodies [415]. Nbs as targets for cancer have been developed against EGFR, HER2, VEGFR2 [415], and continue to be developed for overcoming the immune suppression of the TME and targeting adaptive and innate immune checkpoints amongst other targets [416].

## 9. Conclusions

Deeper understanding of the biology and interactions between the immune system and malignant tumours has enabled the identification of new targets and strategies for immunotherapies. Responses of solid tumours to existing immunotherapies vary and are often short-lived due to the TME that presents a barrier to immune cell infiltration and limits their function. The need for innovative personalized, precision medicine to improve predictability of response and patient survival with less aggressive treatment is still pressing. The characterisation of a patient’s DNA mutations, tumour gene and glycan expression, epigenome, and gut microbiome to stratify them into potential responders or non-responders to immunotherapies will be beneficial. This should optimise the choices and combinations of new and existing immunotherapies and other therapies to overcome the negative influence of the TME and enhance immune responses to the tumours. In addition, for the majority of patients with serous ovarian cancer, the physical barrier and sink for therapeutic antibodies provided by soluble and membrane-bound MUC16, needs to be overcome. This should have the effect of increasing bioavailability of subsequently administered therapies and/or immunotherapies and improve patient response. The continued development of innovative strategies designed to affect the immune system, and their application in combination with other therapies should increasingly improve ovarian cancer patient survival in future.

## Figures and Tables

**Table 1:** Summary of direct and cross-presentation of antigen by professional and non-professional antigen-presenting cells via MHC to T-cells.

	APC Type	MHC Class	Antigen Location	T-Cell Type
Direct Antigen Presentation:	Non-professional APC	MHC I	Intracellular antigen	CD8+
	Professional APC or Tumour cell	MHC II	Extracellular antigen	CD4+
Cross Presentation:	Conventional dendritic cells (cDCs)	MHC I	Extracellular antigen	naïve CD8+

**Table 2:** Immune checkpoints: Binding partner receptor-ligand interactions between antigen-presenting cells or tumour cells, and T-cells or other immune cells, and their downstream effects. Ovarian cancer # and non-ovarian cancer ## expression of ligands is referenced.

APC/Tumour Cell	Immune Cell *	Immune Cell Effect: + Stimulatory; − Inhibitory
MHC class I or II # [30]	TCR	+ (Signal 1)
MHC class II # [30]	LAG-3 (CD223)	-
CD80 (B7-1)	CD28	+ (classic Signal 2)
CD86 (B7-2)	CD28	+ (classic Signal 2)
CD80 (B7-1)	CTLA-4 (CD152)	-
CD86 (B7-2)	CTLA-4 (CD152)	-
PD-L1 (B7-H1, CD274) # [31]	PD-1 (PDCD1/CD279)	-
PD-L2 (B7-DC, CD273) # [31]	PD-1 (PDCD1/CD279)	-
PD-L1 (B7-H1, CD274) #	CD80 (B7-1)	-
HVEM (TNFRSF14) # [32]	BTLA (CD272)	-
B7-H4 (VTCN1) # [33]	B7-H4R	-
ICOSL (B7-H2, ICOSLG) # [34]	ICOS	+
CD137L (TNFSF9)	CD137 (4-1BB, TNFRSF9)	+
CD70 (TNFSF7) # [35]	CD27 (TNFRSF7)	+
GITRL (TNFSF18) ## [36]	CD357 (GITR)	+
OX40L (CD252) # [34]	OX40 (CD134, TNFRSF4)	+
CD40 (TNFRSF5) # [37]	CD40L (CD40LG, CD154, TNFSF5) on NK, T-cells	+
CD47 # [38]	SIRPα on macrophages	-
CD24 # [39]	Siglec-10 on TAMs	-
MUC16 (CA125) # [40]	Siglec-7 on granulocytes, all NK cells	-
MUC1 (CD227) # [41] MUC16 # [40]	Siglec-9 on granulocytes, some NK cells	-
IgG-Fc	CD16 (FcγRIIIA) on NK cells	+
NKG2D ligands # [42,43]	NKG2D on NK cells	+
MHC class I # [30]	KIRs on NK cells	-
MHC class I (HLA-E) # [44]	NKG2A-CD94 on NK cells and CD8+ T-cells	-

* = T-cell unless stated otherwise. (+ stimulatory; -inhibitory). # = ovarian cancer expression. ## = non-ovarian cancer expression.

**Table 3:** Agents with FDA Approval in Clinical Use for Different Cancers, and/or in Clinical Trials for Ovarian Cancer.

Drug	Type	Molecular Target	Target Type	Tumour Types with FDA Approval	Used in Clinical Trials That Include Patients with Ovarian Cancer
T-VEC/Talimogene laherparepvec	Oncolytic virus	GM-CSF encoding	Tumour cells	Melanoma [205]	1 clinical trial for ovarian cancer, [206]
CTL019/tisagenlecleucel	CAR-T	CD19	B-cell marker	Acute lymphoblastic leukaemia; large B-cell lymphoma [207]	NA
Axicabtagene ciloleucel	CAR-T	CD19	B-cell marker	Certain types of large B-cell lymphoma [208]	NA
Blinatumomab	BiTE	CD19, CD3	B-cell marker, TCR	Acute Lymphoblastic Leukaemia [209]	NA
Ipilimumab	mAb	CTLA-4	Checkpoint inhibitor	Melanoma, renal cell carcinoma, colorectal cancer [210]	16 clinical trials for ovarian cancer [211]
Nivolumab	mAb	PD-1	Checkpoint inhibitor	Melanoma, Non-Small Cell Lung Cancer, Renal Cell Carcinoma, Hodgkin’s Lymphoma, Head and Neck Cancer, Urothelial Carcinoma, Colorectal Cancer, Hepatocellular Carcinoma, Small Cell Lung Cancer [212]	29 clinical trials for ovarian cancer [213]
Pembrolizumab	mAb	PD-1	Checkpoint inhibitor	Metastatic Melanoma, Non-Small Cell Lung Cancer, Head and Neck Cancer, Hodgkin’s Lymphoma, Urothelial Carcinoma, Gastric Cancer, Cervical Cancer, Hepatocellular Carcinoma, Merkel Cell Carcinoma, Renal Cell Carcinoma [214]	63 clinical trials for ovarian cancer [215]
Durvalumab	mAb	PD-L1	Checkpoint inhibitor	Urothelial Carcinoma, Non-Small Cell Lung Cancer [216]	26 clinical trials for ovarian cancer [217]
Atezolizumab	mAb	PD-L1	Checkpoint inhibitor	Bladder Cancer, Non-Small Cell Lung Cancer, Breast Cancer, Small Cell Lung Cancer [218]	18 clinical trials for ovarian cancer [219]
Avelumab	mAb	PD-L1	Checkpoint inhibitor	Merkel Cell Carcinoma, Urothelial Carcinoma, Renal Cell Carcinoma [220]	15 clinical trials for ovarian cancer [221]
Relatlimab	mAb	LAG-3	Checkpoint inhibitor	NA (under development for melanoma)	0 clinical trials specifically for ovarian cancer [222], though NCT01968109 includes ovarian cancer patients
Bevacizumab	mAb	VEGF-A	Angiogenesis inhibitor	Colorectal Cancer, Non-Small Cell Lung Cancer, Glioblastoma Multiforme, Renal Cell Carcinoma, Cervical Cancer, Ovarian Cancer, Fallopian Tube Cancer, Peritoneal Cancer [9]	158 clinical trials for ovarian cancer [223]
Hu5F9-G4	mAb	CD47	Phagocytosis regulator	NA (under development for solid tumours and B-cell Non-Hodgkin’s Lymphoma)	1 clinical trial for ovarian cancer NCT03558139 [224].
SGN-CD47M	Antibody-drug conjugate	CD47	Phagocytosis regulator	NA	1 clinical trial for ovarian cancer [225]
Utomilumab	Agonist mAb	CD137 (4-1BB, TNFRSF9)	co-stimulatory molecule	NA	2 clinical trials for ovarian cancer [226]
Varlilumab	Agonist mAb	CD27 (TNFRSF7)	co-stimulatory molecule	NA	3 trials for ovarian cancer [227,228]
Epacadostat	Small molecule	IDO1	Catabolic enzyme	NA	11 clinical trials for ovarian cancer [229]
Daclizumab	mAb	CD25	Tregs	NA, though FDA approval for Multiple Sclerosis [230]	1 clinical trial for ovarian cancer [231]
Ontak (Denileukin diftitox)	Fusion protein	CD25, diphtheria toxin	Tregs	Cutaneous T-cell lymphoma [232]	4 clinical trials for ovarian cancer [233]
Trastuzumab	mAb	HER2	Overexpressed TAA	Breast Cancer, Gastric Cancer [234]	9 clinical trials for ovarian cancer [235]
Pertuzumab	mAb	HER2	Overexpressed TAA	Breast Cancer [236]	6 clinical trials for ovarian cancer [237]
Solitomab (MT110)	BiTE	EpCAM, CD3	Overexpressed TAA, TCR	NA	1 clinical trial for ovarian cancer [238]
Catumaxomab	Trifunctional bi-specific Ab	EpCAM, CD3	Overexpressed TAA, TCR	NA	6 clinical trials for ovarian cancer [239]
Gatipotuzumab (PankoMab-GEX)	mAb	MUC1	Overexpressed TAA	NA	1 clinical trial for ovarian cancer [191,240]
Oregovomab	mAb	CA125/MUC16	Overexpressed TAA	NA	11 clinical trials for ovarian cancer [241], but to date, no benefit derived [242]
Abagovomab	mAb	CA125/MUC16	Overexpressed TAA	NA	3 clinical trials for ovarian cancer [243]
Various	CAR-T	Various, including MSLN	Overexpressed TAA	NA	16 clinical trials for ovarian cancer [244]
CRS-207	*Listeria* vaccine expressing MSLN	MSLN	TAA	NA	2 trials for ovarian cancer [245], including NCT02575807 [246,247]
Amatuximab	mAb	MSLN	Overexpressed TAA	NA	3 clinical trials for ovarian cancer [248]
Anetumab ravtansine	antibody-drug conjugate	MSLN, tubulin	Overexpressed TAA, mitotic cells	NA	2 clinical trials for ovarian cancer [249]
Farletuzumab	mAb	FRα	Overexpressed TAA	NA	7 clinical trials for ovarian cancer [250]
Mirvetuximab soravtansine	antibody-drug conjugate	FRα, tubulin	Overexpressed TAA, mitotic cells	NA	8 clinical trials for ovarian cancer [251]

FDA: US Food and Drug Administration; CAR-T: chimeric antigen receptor modified T-cells; BiTE: bi-specific T-cell engager; TAA: tumour-associated antigens; NA: not applicable.
